# Accessing the electronic structure of liquid crystalline semiconductors with bottom-up electronic coarse-graining†

**DOI:** 10.1039/d3sc06749a

**Published:** 2024-05-02

**Authors:** Chun-I Wang, J. Charlie Maier, Nicholas E. Jackson

**Affiliations:** a Department of Chemistry, University of Illinois at Urbana-Champaign 505 S Mathews Avenue Urbana Illinois 61801 USA jacksonn@illinois.edu

## Abstract

Understanding the relationship between multiscale morphology and electronic structure is a grand challenge for semiconducting soft materials. Computational studies aimed at characterizing these relationships require the complex integration of quantum-chemical (QC) calculations, all-atom and coarse-grained (CG) molecular dynamics simulations, and back-mapping approaches. However, these methods pose substantial computational challenges that limit their application to the requisite length scales of soft material morphologies. Here, we demonstrate the bottom-up electronic coarse-graining (ECG) of morphology-dependent electronic structure in the liquid-crystal-forming semiconductor, 2-(4-methoxyphenyl)-7-octyl-benzothienobenzothiophene (BTBT). ECG is applied to construct density functional theory (DFT)-accurate valence band Hamiltonians of the isotropic and smectic liquid crystal (LC) phases using only the CG representation of BTBT. By bypassing the atomistic resolution and its prohibitive computational costs, ECG enables the first calculations of the morphology dependence of the electronic structure of charge carriers across LC phases at the ∼20 nm length scale, with robust statistical sampling. Kinetic Monte Carlo (kMC) simulations reveal a strong morphology dependence on zero-field charge mobility among different LC phases as well as the presence of two-molecule charge carriers that act as traps and hinder charge transport. We leverage these results to further evaluate the feasibility of developing mesoscopic, field-based ECG models in future works. The fully CG approach to electronic property predictions in LC semiconductors opens a new computational direction for designing electronic processes in soft materials at their characteristic length scales.

## Abstract 2

A bottom-up electronic coarse-graining model predicts morphology-dependent electronic structure and charge transport properties for liquid-crystal semiconductors, without back-mapping or *ad nauseam* quantum chemistry calculations.

## Graphical abstract

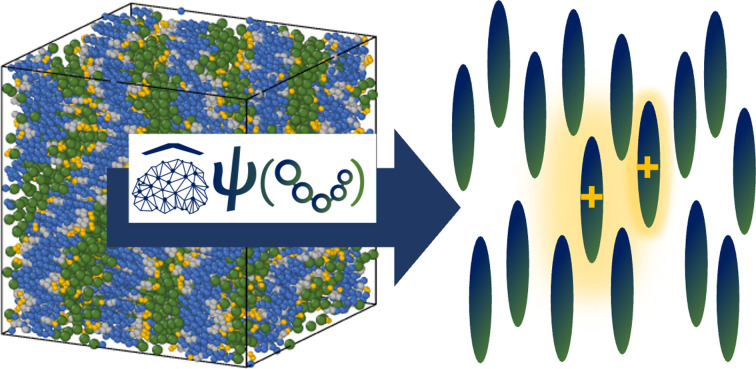

## 1 Introduction

Significant strides have been made in the design of organic semiconductors (OSCs), with diverse applications such as organic light-emitting diodes (OLEDs),^1,2^ organic photovoltaics (OPVs),^3,4^ organic field-effect transistors (OFETs),^5–7^ and biomedical devices.^8–10^ The success of OSCs hinges upon gaining insights into the interplay between optoelectronic properties and multiscale structural attributes, spanning molecular conformations (1–10 Å), primary structural features (1–10 nm), mesoscale morphology (10 nm–10 μm), and thin-film morphology (>10 μm).^11–21^ Multiscale simulations have emerged as an essential tool, shedding light on complex structure–function relationships in OSCs.^22–25^ Traditional multiscale simulations often employ a bottom-up coarse-grained (CG) procedure to model the bulk morphology of OSCs, followed by a series of back-mapping procedures aimed at converting CG coordinates to atomistic resolution.^26–34^ The atomistic coordinates retrieved from the CG resolution facilitate subsequent quantum chemistry (QC) calculations to characterize electronic structure. However, the widespread utilization of multiscale simulations for OSC design has been hampered by the complex workflow, challenges associated with the one-to-many nature of backmapping, and the prohibitive computational cost of QC calculations. While recent efforts have utilized machine learning (ML) approaches to streamline back-mapping,^35–37^ the computation of morphology-dependent electronic properties remains intractable due to the demanding nature of quantum chemistry calculations.

The recent emergence of interest in liquid-crystal-forming semiconductors highlights the computational challenges intrinsic to OSCs. Simply put, the computational challenge of modeling OSCs can be stated as the need to assess quantum-mechanically-derived electronic properties at multiple thermodynamic state points for large (∼10–100 nm), often glassy morphologies with robust statistical sampling.^38–43^ While the manipulation of liquid crystal (LC) phases in OSCs for enhanced device performance is a common theme throughout the community,^44–46^ recent work has explored morphology-dependent conductivity in asymmetric benzothienobenzothiophene-based compounds, a promising type of LC-forming OSCs used in field-effect transistors.^47–66^ These compounds exhibit a remarkable combination of attributes, including high charge mobility, superior solubility and processability, robust thermal durability, and the capability to regulate molecular orientation. While layered smectic phases with head-to-head bilayer (lipid-like) structures have been reported in the asymmetric benzothienobenzothiophene family, recent work by Han *et al.* introduced a derivative, 2-(4-methoxyphenyl)-7-octyl-benzothienobenzothiophene (BTBT),^56,57^ that exhibited a rare nematic phase (long-range order without layered structure) when inserted into rubbed planar anchoring sandwich cells. However, the mechanisms underlying phase transitions between crystals and different smectic phases or the formation of the nematic phase remain unclear.^59–66^ Importantly, large changes in the electronic conductivities were observed in transitioning benzothienobenzothiophene-based molecules through different LC phases. For traditional multiscale computational methods, the analysis of such conductivity trends would represent an effort warranting state-of-the-art computing resources; slow relaxation times would necessitate CG modeling, which would need to be connected with all-atom (AA) backmapping and molecular dynamics, followed by *ad nauseam* QC characterization at the ∼10–100 nm scale to compute morphology dependent electronic properties.

To circumvent the convoluted back-mapping processes and resource-intensive QC calculations, recent efforts have explored the evaluation of quantum mechanical (QM) properties at CG resolutions using a “top-down” approach.^67–71^ This approach leverages anisotropic CG simulations to capture π–π stacking interactions between aromatic moieties and the torsional conformational changes within conjugated backbones. These CG collective variables serve as the basis for estimating structure-dependent electronic properties like electronic coupling using physically-motivated approximations. While these top-down CG approaches have been effective at accessing electronic properties such as charge mobility and molecular orbital delocalization at mesoscopic simulation length scales, they are restricted to simple analytical forms that do not capture the complexity of real chemistries. Additionally, research has highlighted that the collective structural variables based on π–π stacking and backbone torsion alone may not suffice to model structure-dependent electronic properties at the CG level.^72–75^

Recently, data-driven approaches have emerged for the “bottom-up” prediction of electronic properties of soft materials at the CG resolution.^76–84^ These electronic CG (ECG) models leverage ML to establish a mapping from AA electronic structure to CG representation, eliminating the complexities and computational costs associated with back-mapping processes and *ad nauseam* QC. A fundamental insight driving the development of ECG models is the recognition that a single CG configuration encompasses a range of AA configurations, resulting in a “one-to-many” mapping (Fig. S1†). This mapping implies that any property derived from the AA model inherently becomes a probabilistic distribution at the CG resolution. The noise on this distribution can be related to (i) the degeneracy of the CG mapping operator (dictating how atoms are grouped into CG beads) and (ii) the thermodynamic state of the system (reflecting the extent of thermal fluctuations within the AA model). Recently, our group has extended the ECG framework through the incorporation of deep kernel learning (DKL) with approximate Gaussian processes^81,82^ to predict noisy, heteroscedastic distributions as a function of CG representation, facilitating the rigorous bottom-up connection of ECG predictions with an underlying, QC-accurate AA model.

In this work, we demonstrate the first bottom-up CG study of the bulk electronic structure of the molecular semiconductor BTBT,^55–58^ as a function of the LC morphology using ECG methods. Our comprehensive structural characterization of the simulations reveal the presence of smectic A and smectic E phases, instead of the experimentally observed nematic phase under planar anchoring conditions, which suggests a general preference for the benzothienobenzothiophene family to exhibit smectic characteristics. We further explore the dependence of charge delocalization on LC morphology by explicitly constructing density functional theory (DFT)-quality electronic Hamiltonians using only the CG model resolution, from which electronic structure at the ∼20 nm length scale is derived with statistical robustness. Analyses of these Hamiltonians reveal the presence of three distinct types of charge carriers, the distributions of which vary between LC phases. We trace the origin of these charge carriers to multi-molecule descriptors of local electronic and structural environments. The zero-field charge mobility across LC phases, as determined by kinetic Monte Carlo (kMC) simulations, not only underscores a pronounced dependence on morphology but also indicates that two-molecule charge carriers serve as traps, impeding effective charge transport pathways. As ECG methods exhibit approximately 10^5^ reduced cost relative to existing multiscale computational paradigms, a statistically robust characterization of the electronic structure of LC phases at large-length scales is achieved without invoking vast computational resources. Lastly, we examine the potential for connecting bottom-up CG predictions of electronic structure with field-based order parameters that serve as the workhorses of the soft materials theory community. Altogether, this fully “bottom-up” approach to morphology and electronic structure calculations facilitates the systematic design of OSCs across diverse morphologies at mesoscopic length scales with statistical robustness and low computational cost.

## 2 Methods

**Fig. 1:**
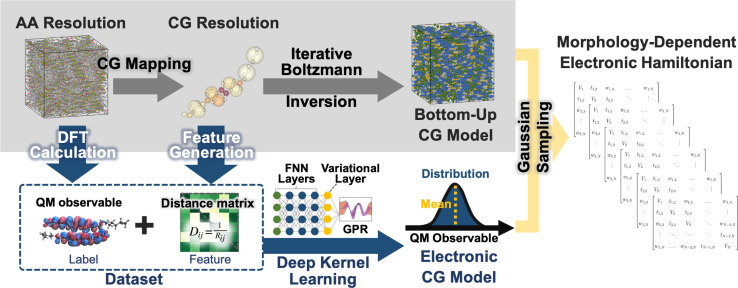
Workflow depicting the bottom-up CG models (top-left panel), ECG models (bottom-left panel), and the morphology-dependent electronic Hamiltonian (right panel). Gray arrows illustrate the bottom-up CG model development process. Blue arrows outline the workflow, encompassing data set creation, ML model training, and the prediction of electronic observables for a given CG configuration. The inset illustrates the architecture of deep kernel learning (DKL), incorporating a feed-forward neural network (FNN), a variational layer, and Gaussian process regression (GPR). The orange arrow signifies that Hamiltonians are sampled from the ECG-predicted Gaussian distribution.

The workflow for the fully bottom-up CG method for the LC phases of BTBT is illustrated in Fig. 1. AA molecular dynamics (MD) simulations are performed in the isotropic (700 K and 1 bar), smectic A (555 K and 1 bar),‡‡Both smectic phases exhibit a layered structure, but in the smectic A phase, the primary director and layer normal are parallel, whereas in the smectic E phase, there is a tilt angle between them, along with a unit pattern among the layer structure. A detailed structural characterization of various LC phases is provided in the subsequent section. and smectic E (515 K and 1 bar)‡ phases to parameterize CG models using iterative Boltzmann inversion (IBI)^85,86^ and an electronic structure-optimized CG mapping.^87^ CG structural prediction models are coupled with bottom-up ECG models derived from AA MD trajectories and ωB97XD/cc-pVDZ DFT calculations. CG representations were converted to CG distance matrices, providing translational and rotational invariance, that served as input features for the DKL method underlying ECG. ECG models were trained to reproduce (i) conformation-dependent, single-molecule highest occupied molecular orbital (HOMO) energies of BTBT as well as (ii) HOMO–HOMO electronic couplings between BTBT dimers. Comprehensive details regarding the parameterization and validation of the AA force field and the creation of BTBT training data sets can be found in previous work.^87^

### 2.1 Bottom-up CG models for morphology prediction

Iterative Boltzmann Inversion (IBI)^85,86^ with pressure correction^88^ is used to construct CG intermolecular potentials for BTBT in the isotropic and smectic phases. IBI was selected due to the importance of structural prediction accuracy in ECG that was recently reported,^80^ though in principle more rigorous bottom-up CG methods can be utilized as long as structural distribution functions are accurately reproduced.

As CG non-bonded potentials are expected to exhibit limited thermodynamic transferability between isotropic and smectic phases, two CG non-bonded potentials were developed for the isotropic (700 K and 1 bar) and smectic A phases (at 555 K and 1 bar), respectively. Achieving transferability in bottom-up CG necessitates a similarity in the effective interaction domain, such as isotropic to isotropic or anisotropic to anisotropic, rather than transitioning from isotropic to anisotropic phases. This requirement arises from the collective interaction of mapped atoms, encapsulated by an effective CG potential of mean force, which in turn represents the corresponding free energy attributes.^89–92^ Therefore, for the smectic E phase (at 515 K and 1 bar), the CG non-bonded potentials were adopted from those designed for the smectic A phase, which demonstrated excellent transferability in reproducing both short-range and long-range structural properties, as discussed in the ESI.† A single set of CG bonded potentials of BTBT, encompassing CG bond, CG angle, and CG dihedral interactions, were determined through direct Boltzmann inversion using the reference AA isotropic morphology; previous works have demonstrated the low sensitivity of CG bonded parameters to thermodynamic state changes.^86,93,94^ All AA and CG MD simulations were conducted using LAMMPS,^95^ with a custom IBI implementation adhering to established procedures.^96–98^ Additional details regarding the construction of CG structural prediction models and a comprehensive evaluation including the radial distribution function (RDFs) of the center of mass, nematic order parameters, structure factor analyses, and RDFs of the 120 distinctive CG pairs are available in the ESI.†

While the IBI methodology and its derivatives have been extensively utilized in the development of bottom-up CG models for liquid-crystal-forming materials,^99–111^ we observed significant limitations in its application to the complex intermolecular interactions of BTBT. Specifically, the extensive fused ring motifs of BTBT, coupled with the flexible alkyl side chain, induce strongly anisotropic intermolecular interactions. This asymmetric molecular architecture poses challenges for the convergence of the IBI procedure, particularly in high-order LC phases. The presence of a rigid fused ring, a floppy alkyl side chain, along with the highly asymmetric structure necessitates the treatment of all 15 CG particles as distinct types. This results in the requirement for 120 CG non-bonded potentials, further complicating the challenge of the IBI parameterization. We observed that IBI convergence for BTBT in the smectic A phase was slow and extremely sensitive to initializations and damping factors compared to the isotropic phase. Although the CG non-bonded potentials derived for the smectic A phase exhibit excellent transferability to the smectic E phase, we attempted to further refine the smectic E CG non-bonded potentials. Unfortunately, the convergence of the IBI approach proved intractable in this more anisotropic system. We note that the development of CG potentials for BTBT or similar fused-ring materials^112,113^ in condensed phases is uncommon in the literature to-date. Recent research on anisotropic CG models^114–122^ or ML-derived CG potentials^123,124^ has explored their potential to accommodate the strong anisotropy of the polycyclic aromatic hydrocarbon systems, but this topic falls beyond the scope of the present work.

### 2.2 Development of electronic (ECG) models

We employ the DKL version of ECG^81^ to create CG electronic predictions models that capture configurational variations of BTBT's HOMO energy as well as the HOMO–HOMO electronic coupling between BTBT dimers, eliminating the need for backmapping or *ad nauseam* QC. The DKL-ECG model consists of a feed-forward neural network (FNN), a variational layer, and Gaussian process regression (GPR), as illustrated in the inset of Fig. 1. The FNN transforms CG conformations (distance matrices) followed by a variational layer that maps the FNN results to a latent space. From this latent space, approximate GPR is used to map to the electronic prediction task. Critically, DKL-ECG allows the noisy observables at the CG resolution to be treated as heteroscedastic Gaussian distributions, with predicted means and widths varying as functions of positions in CG configuration space. Accurately representing these CG probability distributions of electronic properties is essential to achieving an accurate “bottom-up” reproduction of the electronic structure of the system. Stochastic sampling of these Gaussian distributions as a function of CG configuration allows the reproduction of the correction AA ensemble of DFT-quality electronic structure without the need for backmapping or *ad nauseam* QC, providing the critical computational cost advantage without loss of accuracy. For in-depth methodological details of DKL-ECG, the reader is referred to ref. 81.

Training sets for DKL-ECG models are obtained from previous AA simulations of BTBT.^87^ These trajectories encompass isotropic and smectic morphologies, followed by electronic structure calculations of single molecule and dimer pairs at the ωB97XD/cc-pVDZ level of theory. In subsequent discussions, we refer to the data set extracted from the isotropic morphology as training-isotropic/testing-isotropic, and the data set originating from the smectic A morphology as training-smectic A/testing-smectic A. Comprehensive details regarding the training procedure of the DKL-ECG models for HOMO energy prediction can be found in the ESI.† Training ML models for predicting electronic coupling has consistently posed a formidable challenge, even when including full AA featurization.^125–128^ This challenge stems from the electronic coupling's intricate dependence on both the separation distance and mutual orientation between molecular pairs.^11,129^ To address this challenge, we explored many approaches (see ESI†) and settled on learning the logarithm of the absolute value of the electronic coupling combined with a phase classifier that predicts the sign of the HOMO–HOMO electronic coupling for a given CG molecular pair conformation using a FNN. Detailed information on the model parameters and the training procedures for the DKL-ECG models and the FNN classifier can be found in the ESI.†

### 2.3 Valence Hamiltonian construction at the CG resolution

We integrate CG structural prediction models and ECG models to construct tight-binding Hamiltonians for the isotropic and smectic phases in a basis of HOMO orbitals using only the CG representation of BTBT. 9000 BTBT molecules are simulated at the CG resolution in the isotropic and smectic LC phases within cubic boxes measuring 199 Å, 190 Å, and 188 Å, respectively. Ten replicas of each CG simulation (40 ns) were performed for each phase. 120 snapshots of the CG configuration of the system were collected from each simulation. These snapshots served to define the morphology-dependent electronic Hamiltonian at the CG level.^130–132^1
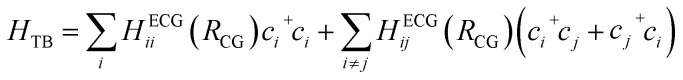


A tight-binding Hamiltonian in the basis of BTBT's HOMO orbitals was employed to model the valence band electronic structure relevant to hole transport in BTBT morphologies (eqn (1)), where *H*^ECG^_*ii*_(*R*_CG_) and *H*^ECG^_*ij*_(*R*_CG_) represent the on-site ECG-derived HOMO energy prediction and dimer HOMO–HOMO electronic coupling predictions, respectively. Provided the single-molecule and dimer configurations extracted from the CG simulations, ECG models predict the Gaussian means and variances of the HOMO energy and HOMO–HOMO electronic coupling as illustrated in Fig. 1 resulting in population of all matrix elements of the Hamiltonian. For each single-molecule CG configuration, we performed Gaussian sampling using the predicted mean and variance, generating 20 distinct samples of the HOMO energies that served as the individual diagonal elements of 20 unique Hamiltonians. The off-diagonal elements of these Hamiltonians underwent the same Gaussian sampling process, with the coupling value set to zero when the distance between the center of mass (COM) of the pair molecules exceeded 7 Å. This sampling process explicitly addresses the one-to-many mapping influenced by thermodynamic fluctuations and mapping degeneracy, reproducing the correct AA-ensemble of thermodynamically averaged electronic predictions without backmapping. Subsequently, these ECG-determined Hamiltonians were diagonalized to compute the delocalized electronic states in the isotropic and smectic LC phases, from which subsequent analysis occurred.

The ECG approach to construct electronic tight-binding Hamiltonians at the CG resolution exhibits dramatic computational cost benefits relative to traditional multiscale modeling paradigms. Establishing an individual Hamiltonian using this method is conservatively estimated to be at least 10^5^ times faster than equivalent DFT calculations, and this estimation does not account for the computational cost of backmapping procedures or additional sampling with AA MD. In this study, the task of creating a single Hamiltonian involved handling 9000 single-molecule configurations and roughly 10 000 molecular pairs (within a COM distance of 7 Å) for the evaluation of HOMO energy and electronic coupling, respectively. To characterize the electronic structure of 7200 Hamiltonians with *ad nauseam* DFT calculations at the *ω*B97XD/cc-pVDZ level, executed on a single CPU core, would require approximately 83 520 000 CPU hours (∼7.2 × 10^7^ individual calculations). In contrast, the DKL-ECG method accomplishes this computation in just 107 CPU hours. Relative to the computational cost of training set generation for all ECG models used in this work (cumulatively ∼7.5 × 10^5^ DFT calculations), this amounts to a two order of magnitude advantage that only improves with additional statistical sampling and application of the trained ECG models. This outstanding computational efficiency empowers us to generate 2400 Hamiltonians (comprising 120 CG snapshots multiplied by 20 rounds of Gaussian sampling) for each LC system. This extensive characterization provides a comprehensive and statistically significant understanding of electronic structure in the LC phases, a level of detail that was previously beyond the reach of traditional multiscale simulation methods due to intractable computational costs.

## 3 Results and discussion

In this work, we employ bottom-up CG models to study the morphology of a system of 9000 BTBT molecules at temperatures of 700 K, 555 K, and 515 K, followed by the assessment of ECG models for predicting HOMO energy and HOMO–HOMO electronic coupling. Subsequently, we systematically explore the relationship between different LC structures and their charge transport properties by integrating the bottom-up CG approach and ECG models.

### 3.1 Morphology characterization

**Fig. 2:**
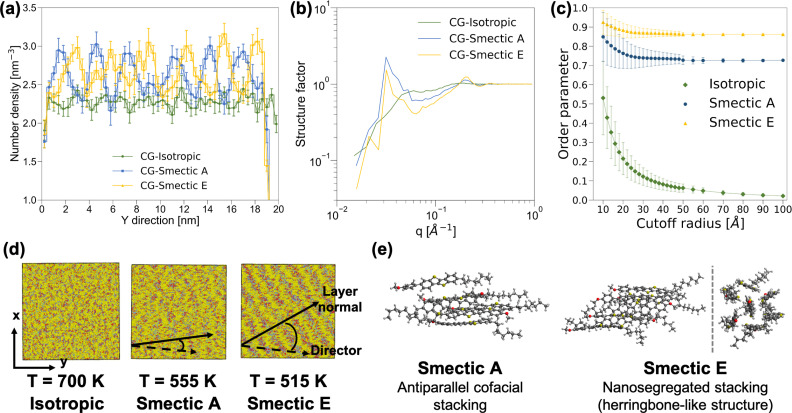
Structural characterization of the CG isotropic, smectic A, and smectic E phases: (a) number density distribution of the center of mass along *y*-axis, (b) structure factor distributions, (c) nematic order parameters of the molecular long-axis as a function of characterization radius, (d) snapshots of CG morphology, where the dashed line and solid line represent the principal director and the layer normal, respectively, and (e) representative snapshots of BTBT local packing structure within the smectic layers extracted from AA MD simulations. The analyses depicted in (a)–(d) are conducted on a system comprising 9000 BTBT molecules across the three specified temperatures. The molecular long-axis for the nematic order parameter analysis is determined by the moment of inertia of each BTBT molecule at CG resolution.

Based on the observation of differential scanning calorimetry with the insertion of BTBT into rubbed planar anchoring sandwich cells,^56,57^ our simulation should yield distinct phases at temperatures of 700 K, 555 K, and 515 K, corresponding to isotropic, nematic, and smectic A phases, respectively. The structural characterization of the morphology at 700 K, as depicted in Fig. 2, confirms the anticipated isotropic features. However, an unexpected discovery emerged when clear layered structures were observed in both AA and CG simulations at 555 K and 515 K, meeting the key criteria for distinguishing smectic phases from the nematic phase. Fig. 2a and b reveal oscillating patterns in number density along the *y*-axis direction and a distinct peak at *q* = 0.03 Å^−1^ in the structure factor analyses,§§The structure factor analysis in this study is based on the Fourier transform of particle density averaged across the three Cartesian coordinates. Due to the inherent anisotropic nature of both smectic phases, we evaluated the structure factor separately along each Cartesian axis and then normalized the results across all three axes. indicating smectic phases at 555 K and 515 K. An additional AA MD simulation was performed with the system temperature linearly decreasing at a rate of 3.5 K ns^−1^ from 700 K to 350 K. As illustrated in Fig. S8,† we observed changes in the slope of the nematic order parameters for both the molecular long-axis and short-axis around 640 K, and another change in the slope of the order parameter of the molecular short-axis around 520 K. Fig. S8† also shows a clear change in the structure factor peak at *q* = 0.03 Å^−1^, corresponding a layered structure of period 33 Å, at both phase transition temperatures. The persistent presence of layered-structure below 640 K in the structure factor analysis suggests the absence of the nematic phase in our simulations. Given that the major difference between BTBT and the well-studied benzothienobenzothiophene family is the methoxy group at the tail of the phenyl ring, we suspect that the nematic phase can only be achieved under specific boundary conditions, such as those provided by the rubbed planar anchoring sandwich cells.^56,57^

Further analyses were conducted to differentiate between the two smectic phases at 555 K and 515 K. The nematic order parameter analysis revealed that the CG morphology at 555 K exhibited an order parameter of 0.733 ± 0.008 along with a principal director vector (−0.093 ± 0.009, 0.994 ± 0.001, −0.048 ± 0.012), and a tilt angle between the principal director vector and layer normal of 17.1 ± 16.0°. The similar mean value and standard deviation of the tilt angle imply parallel alignment and suggest a smectic A phase. Conversely, for the 515 K CG morphology, a larger order parameter of 0.867 ± 0.004 was observed along with a principal director vector (−0.114 ± 0.003, 0.986 ± 0.002, −0.123 ± 0.010), and a clear deviation between the principal director vector and layer normal with the tilt angle of 34.1 ± 12.2°, as shown in Fig. 2d, indicates a smectic E phase. In addition, snapshots extracted from AA MD simulations at 555 K and 515 K illustrate the differences between smectic A and smectic E phases. While BTBT molecules form antiparallel cofacial π–π stacking in the smectic A phase, a herringbone-like structure with offset π–π stacking is observed in the smectic E phase, reminiscent of the crystal structure observed in the benzothienobenzothiophene family.^47–55,59–66^ These features are consistent with CG structure factor analyses and the characterization of the nematic order parameter as a function of cutoff radius, as depicted in Fig. 2b and c. Specifically, the smectic E phase exhibits a stronger peak at *q* = 0.21 Å^−1^ and a larger order parameter with smaller standard deviation, reflecting more ordered π–π stacking in the herringbone-like structure. The decreasing peak intensity at *q* around 0.03 Å^−1^ and its shift to the low *q* regime indicates offset π–π stacking monolayer structure.

The observation of monolayers with a length scale around 33 Å, featuring antiparallel π–π stacking within these layered structures, provides insights into the phase transition mechanisms between smectic and crystal phases. Since the initial use of asymmetric benzothienobenzothiophene in field-effect transistors, a head-to-head bilayer (lipid-like) unit structure in the crystal phase has consistently been reported. It was conventionally believed that the head-to-head bilayer should be a common feature in smectic phases as well.^47–55^ However, recent studies have presented more evidence of monolayer structures along with antiparallel π–π stacking in smectic phases. Our simulation results align with these recent findings.^59–66^ Specifically, the transition from antiparallel cofacial π–π stacking^133^ to offset π–π stacking (also known as nanosegregated stacking)^134,135^ within the monolayers from smectic A to smectic E, accompanied by a considerable increase in the tilt angle between the principal director and the layer normal, may serve as a precursor to the formation of the lipid-like bilayer structure observed in the crystal phase.^65,66^

### 3.2 Evaluation of ECG models

We first scrutinize the performance of the DKL-ECG model in predicting the HOMO energy and HOMO–HOMO electronic coupling as inputs for constructing the tight-binding Hamiltonian (eqn (1)).

#### 3.2.1 HOMO energy prediction

**Fig. 3:**
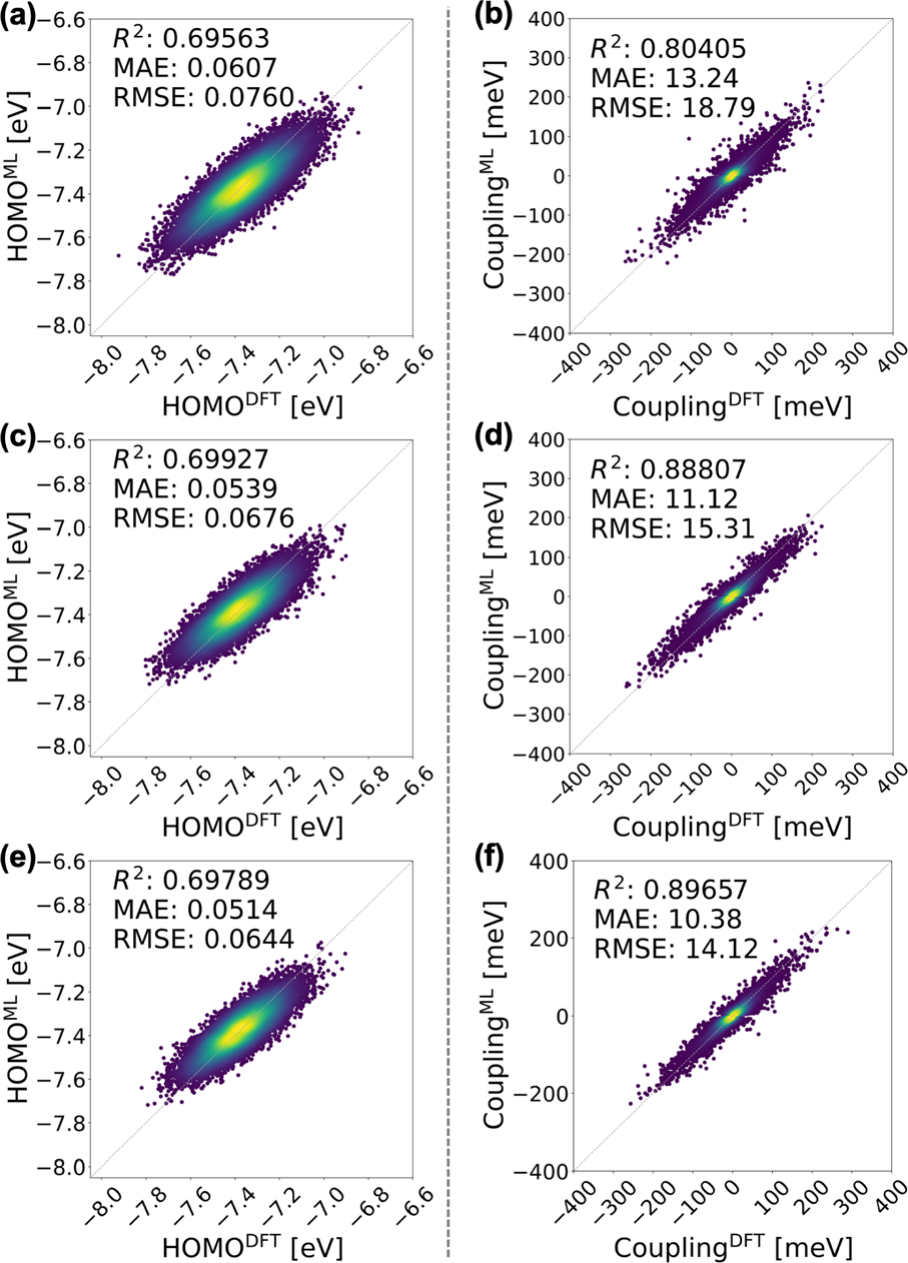
Evaluation of the DKL-ECG models for predicting HOMO energy (left panels) and HOMO–HOMO electronic coupling (right panels). The mean values of HOMO energy predicted by the DKL-ECG models are compared with values obtained through DFT calculations across the (a) testing-isotropic, (c) testing-smectic A, and (e) testing-smectic E data sets. The right panels assess HOMO–HOMO electronic coupling predictions, utilizing DFT calculations as benchmarks, and contrasting values derived from the DKL-predicted mean and a FNN sign classifier, for the (b) testing-isotropic (d) testing-smectic A, and (f) testing-smectic E data sets. The heatmap visually represents the density of data points. The DKL-ECG model for HOMO energy prediction was trained on the training-isotropic data set. ECG for electronic coupling predictions are derived from the DKL regression model trained on the training-smectic A data set and the FNN classification model trained on the training-isotropic data set. Detailed evaluations of these ECG models are discussed in the ESI.†

As we are employing a CG representation optimized to the task of HOMO-related prediction tasks,^87^ the ECG model for HOMO energy prediction exhibits high accuracy in the isotropic and smectic LC phases of BTBT. As depicted in Fig. 3, the *R*^2^ values between DFT ground-truths and ECG-predicted means consistently hovered around 0.7 with the mean absolute error (MAE) around 0.06 eV. In Fig. S9 and S10,† the ensemble averages from the ECG models consistently aligned with the distributions of both training and testing data sets. Fig. 3 further highlights the ECG model's transferability from the isotropic data set (training-isotropic) to both smectic data sets (testing-smectic A and testing-smectic E). It is critical to note that a *R*^2^ value <1 is not indicative of poor performance of the ECG model; as the prediction task occurs at the CG resolution, there is an intrinsic “noise” on the electronic prediction task that limits the computed *R*^2^ value for the mean prediction from DKL. However, as shown in previous work, DKL can accurately reproduce the probability distribution of predicted values at each CG configuration, which renders the effective *R*^2^, in the limit of stochastic Gaussian sampling, much higher. The transferability of the DKL-ECG model can be attributed to the broader configuration space covered by the isotropic data set, as validated by a principal component analysis (PCA) conducted on both data sets, details of which are provided in the ESI.†

#### 3.2.2 HOMO–HOMO electronic coupling prediction

As depicted in Fig. 3, the combined ECG model exhibits robust predictive capabilities for HOMO–HOMO electronic coupling, yielding *R*^2^ and MAE values of approximately 0.89 (0.80) and 11 (13) meV, respectively, for the smectic A (isotropic) data sets. A thorough assessment of ECG models in both LC phases is discussed in the ESI.† It is noteworthy that the FNN classifier correctly predicts the sign of large-value (>100 meV) coupling data with a small number of misclassifications in the second and fourth quadrants of Fig. 3, suggesting potential errors in regions where couplings switch signs, as anticipated. Moreover, Fig. 3 highlights the accuracy of ECG predictions for the smectic and isotropic data sets. When comparing the performance of DKL models between HOMO energy and electronic coupling prediction, the models for coupling consistently outperform those for HOMO energy. This trend can be attributed to the distinction between intramolecular and intermolecular properties in this ML task. HOMO energy is an intramolecular property, and its dependence on a single molecular conformation leads to a significant loss of information at the CG level. Our results agree with previous studies highlighting the sensitivity of single-molecule electronic properties to CG resolution.^80,81^ In contrast, the coupling value signifies the overlap of molecular orbitals between two molecules, and information regarding distance and orientation, especially for the conjugated moiety, is well-preserved at the current CG level.

It is crucial to underscore the significantly reduced computational cost offered by the DKL-ECG approach. The DKL-ECG predicts the HOMO–HOMO coupling of 10 000 molecular pairs in 80 CPU seconds at single-time execution, while DFT calculations demand 4200 CPU seconds for a single pair, equating to 11 600 CPU hours for 10 000 molecular pairs with repeating executions. The computational efficiency of ECG models empowers the establishment of over 4000 morphology-dependent electronic Hamiltonians in this work, facilitating an in-depth exploration of bulk electronic structure with robust statistical sampling.

### 3.3 Characterization of electronic structure in the isotropic and smectic phases

The combination of bottom-up CG structural prediction models with ECG models enables the generation of electronic Hamiltonians at the scale of ∼20 nm that can be used to explore the interplay between morphology and electronic structure in the LC phases of BTBT. We first employed bottom-up CG models to simulate 9000 BTBT molecules in the isotropic and smectic phases. The resulting ensemble-averaged nematic order parameters are 0.018 ± 0.005, 0.733 ± 0.008, and 0.867 ± 0.004 for the isotropic, smectic A, and smectic E phases, respectively. Subsequently, we sampled 2400 electronic Hamiltonians for each LC phase based on ECG predictions derived from the collected snapshots of CG configurations. The diagonalization of these Hamiltonians provided eigenstates (*E*_*j*_) and eigenvectors 
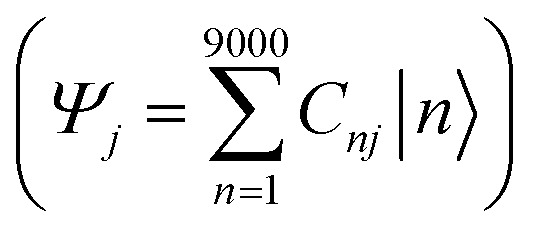
, where *E*_*j*_ represents the energy of the *j*_th_ eigenstate, and *C*_*nj*_ is the wave function coefficient of the *n*_th_ BTBT molecule for the *j*_th_ eigenstate. Subsequently, we computed the inverse participation ratio (IPR) defined as 
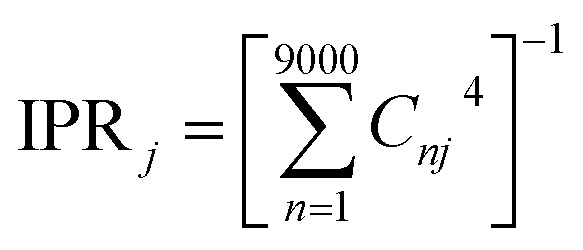
, to quantify the delocalization of the wavefunction throughout the LC phases.

**Fig. 4:**
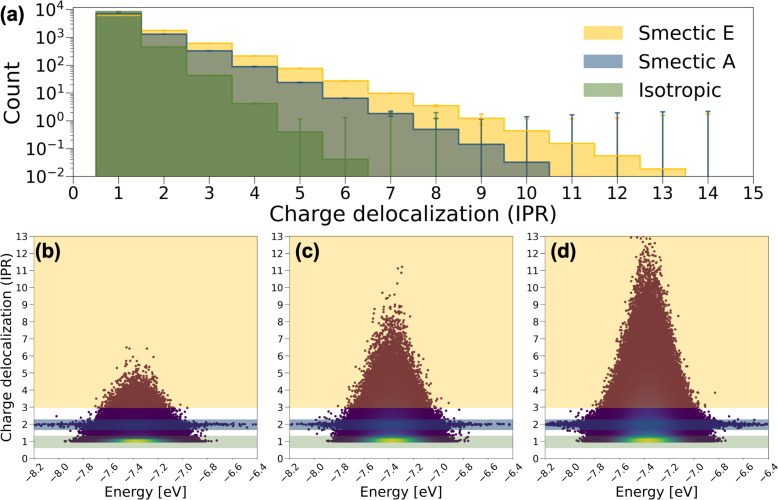
(a) Histograms of the number of charge delocalized molecules (IPR value), and the corresponding IPR value distribution plotted against CT state energy across 9000 CT states for (b) the isotropic phase, (c) smectic A phase, and (d) smectic E phase. Orange, blue, and green masks highlight CT states with IPR > 3, IPR ∼ 2, and IPR ∼ 1, respectively. The heatmap provides a visual representation of the data point density. The histograms represent averages over the 2400 Hamiltonians for each LC phase.

In Fig. 4a, the averaged histogram of the number of charge-delocalized molecules (IPR) over the 9000 eigenstates reveals that charge carriers are predominantly localized on one or two molecules in all LC phases. However, in smectic morphologies, there is a notable increase in the presence of delocalized states, allowing charge carriers to extend across 6 to 10 BTBT molecules for smectic A and 6 to 13 BTBT molecules for smectic E. This observation aligns with experimental findings indicating enhanced mobility with increased ordering of the LC phases in BTBT.^56–58^ It is noteworthy that among the IPR analyses derived from the 2400 Hamiltonians for smectic morphologies, a small number of Hamiltonians do not exhibit the 6 to 13-molecule charge delocalization observed in the majority of cases. Such statistical fluctuations can be critical in soft materials theory, and our effort provides the first robust analysis of the ensemble averaged electronic properties of soft materials, ensuring reliable insights.

To further understand the electronic structure of BTBT in each LC phase, the IPR values for the 9000 charge transport (CT) states derived from each Hamiltonian are depicted as a function of their energies in Fig. 4b–d. Notably, a pronounced concentration of CT states is observed in the IPR ∼ 1 region, particularly prominent in the isotropic and smectic phases. Furthermore, a consistent pattern in the IPR ∼ 2 region is identified across each LC phase, where CT energies span a broad range from −8.2 to −6.4 eV. These distinctive patterns serve as the basis for categorizing CT states into “one-molecule charge carriers” and “two-molecule charge carriers,” while CT states that delocalize over more than three molecules are labeled as “delocalized charge carriers.” To further dissect structural and electronic contributions to the appearance of these charge carrier motifs, we recalculated and diagonalized the Hamiltonians for all systems by setting all diagonal elements to the mean value of HOMO energy across all molecules in the training set (site-energy disorder equal to zero). As shown in Fig. S17,† elimination of site energy disorder (implying identical conformations for all BTBT molecules) facilitates dramatically increased delocalization across 10 to 3000 molecules in 9% of CT states for both isotropic and smectic A phases, and 4.3% for the smectic phase. This result is important for the field of LC material modeling as nearly all simulation approaches employ anisotropic ellipsoids or field-based descriptors, for which such molecule-specific energetic disorder is absent. Moreover, HOMO energy disorder is observed to be smaller in the smectic E phase followed by the smectic A and the isotropic phase, which likely drives the increased charge delocalization. Interestingly, the pattern of two-molecule charge carriers remains unchanged by the manipulation of on-site energy disorder, implying a more detailed interplay of structural and electronic properties in charge delocalization.

**Fig. 5:**
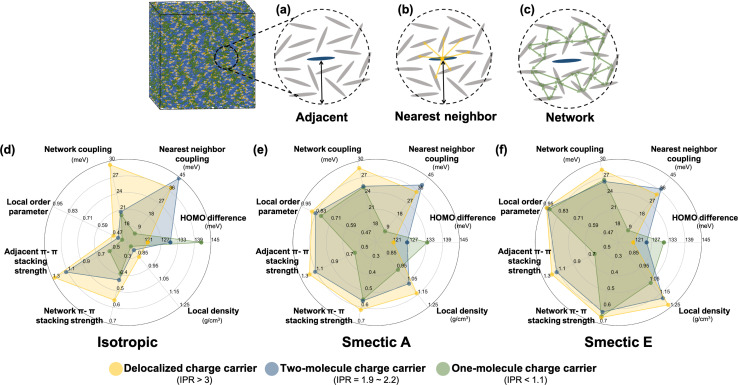
Schematic representations detailing the statistical analyses of structural and electronic features related to (a) all molecules adjacent to the charge center within the characteristic radius, (b) nearest neighbor molecules surrounding the charge center, and (c) the molecular network excluding the charge center within the characteristic radius. Radar charts depict the mean values of the structural and electronic features in the (d) isotropic, (e) smectic A, and (f) smectic E phase. The characteristic radius of 13 Å is chosen to achieve two objectives: first, to allow for the quantification of network properties like network coupling and network π–π-stacking strength, which require a minimum of two molecular shells for statistically significant analysis; second, to accommodate the variability in the size of the first molecular shell observed across different LC phases.

To further investigate the formation of the three primary charge carrier types, we conducted a comprehensive structural characterization based on the local molecular environment of the charge carrier. In the subsequent analyses, to minimize uncertainties arising from CG representation or thermal fluctuations, charge carriers were categorized based on their IPR values: one-molecule charge carriers with IPR values below 1.1, two-molecule charge carriers with IPR values ranging from 1.9 to 2.2, and delocalized charge carriers with IPR values larger than 3. For each charge carrier type, the charge center of the *j*_th_ electronic state was determined by the center of mass of the molecule possessing the largest *C*_*nj*_ coefficient. We included *N*_adj_ adjacent molecules within a 13 Å radius, corresponding to the second molecular shell determined by the RDFs shown in Fig. S2b,† to estimate adjacent structural and electronic characteristics, as illustrated in Fig. 5a–c. These properties included the averaged absolute value of HOMO energy differences between the charge center and all adjacent molecules, local order parameters derived from the nematic order tensor,^38^ the local density, the averaged absolute value of the electronic coupling between the charge center molecule and its nearest neighbor (NN) molecules, the network coupling, representing the averaged absolute value of the electronic coupling between molecules within the cutoff radius (13 Å), and the averaged adjacent π–π stacking strength^68^ between the charge center molecules and adjacent molecules defined by eqn (2):2
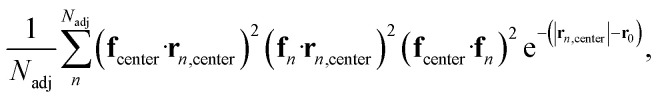
where the vectors **f**_center_ and **f**_*n*_ represent the normal vectors of the conjugated moiety of the charge center BTBT and its *n*_th_ adjacent BTBT, respectively. **r**_*n*,center_ denotes the center-of-mass vector between the BTBT pair. A value of 3.5 (Å) for *r*_0_ ensures the optimal π–π stacking strength at the most ideal π–π stacking distance in the CG model. The average adjacent π–π stacking strength quantifies the extent of π–π stacking between the reference molecule and its neighboring molecules, considering both their separation distance and mutual orientation.

In Fig. 5d–f, the radar plots depict the mean values of all computed electronic descriptors for the three types of charge carriers. These mean values are averaged over the results obtained from 2400 Hamiltonians for the isotropic and smectic phases, respectively. One-molecule charge carriers exhibit the largest HOMO energy difference (141 meV isotropic, 133 meV smectic A, 131 meV smectic E) but the lowest nearest neighbor coupling (6 meV isotropic, 8 meV both smectic phases), as well as the smallest network coupling (20 meV isotropic, 25 meV smectic A). In contrast, delocalized charge carriers exhibit the smallest HOMO energy difference (121 meV isotropic, 121 meV smectic A, 120 meV smectic E) but the largest network coupling (29 meV isotropic, 29 meV smectic A, 28.5 meV smectic E). These findings imply that an effective charge transport network, characterized by strong network coupling, moderate nearest neighbor coupling, and low onsite energy disorder, facilitates charge delocalization. Two-molecule charge carriers demonstrate the strongest nearest neighbor coupling (44 meV isotropic, 39 meV smectic A, 37 meV smectic E) among the three classes, surpassing even their network coupling (21 meV isotropic, 25 meV smectic A, 26 meV smectic E). This result suggests that a stronger nearest neighbor coupling compared to network coupling can result in the localization of charge carriers onto two molecules, which may act as a trap during charge transport. Notably, the prevalence of two-molecule charge carriers aligns with the results of quantum dynamics simulations crystalline organic semiconductors^136–138^ and the model framework of transient localization theory.^139–143^ As LCs are intermediate between isotropic and crystalline systems, the presence of such electronic states is of crucial importance for understanding charge carrier transport in LC materials.

The three types of charge carriers directly reflect their local structural environments. As depicted in Fig. 5d–f, delocalized charge carriers in the isotropic and smectic phases exhibit large values of the local nematic order parameter, local density, and adjacent and network π–π stacking strengths. In contrast, one-molecule charge carriers display the lowest values for all structural features, particularly in terms of adjacent π–π stacking strength. In both smectic phase, even though one-molecule charge carriers demonstrate a similar level of network π–π stacking strength, their small adjacent π–π stacking strength indicates a poor connection between nearest neighbors that substantially impedes charge delocalization. Notably, the large nearest neighbor coupling observed in two-molecule charge carriers does not necessarily correlate with adjacent π–π stacking strength, as only a single molecular pair has a large π–π stacking strength. This result suggests that averaged structural characterizations can wash out the contribution of critical local molecular aggregates even at the 1–10 nm length scale.

### 3.4 Correlation between morphology and charge mobility

To explore the variation in charge mobility across different LC morphologies and understand the impact of the three identified charge carriers on the charge transport mechanism, we conducted rejection-free kMC simulations to estimate the zero-field charge mobility following the methodology outlined in ref. 144–146 (details provided in the ESI†). For each LC phase, kMC charge hopping trajectories were run for 10^5^ steps based on 100 CG configurations/Hamiltonians, utilizing 9000 molecules as different initial hopping sites, resulting in 900 000 kMC trajectories per LC phase. Analysis of the kMC trajectories with different initial hopping sites revealed that in the isotropic phase, 65.0 ± 1.0% of the initial sites exhibited zero mobility, indicating that the charge carriers were trapped near these initial sites within 10^5^ steps, attributed to either one-molecule or two-molecule charge localized carrier states. Furthermore, the partition of zero mobility due to the localized initial charge carriers in the smectic A and smectic E phases was 35.4 ± 1.2% and 17.9 ± 1.2%, respectively, indicating the inability of these carriers to establish effective charge transport pathways in all phases, but with a portion that decreased as a function of increase LC ordering.

**Fig. 6:**
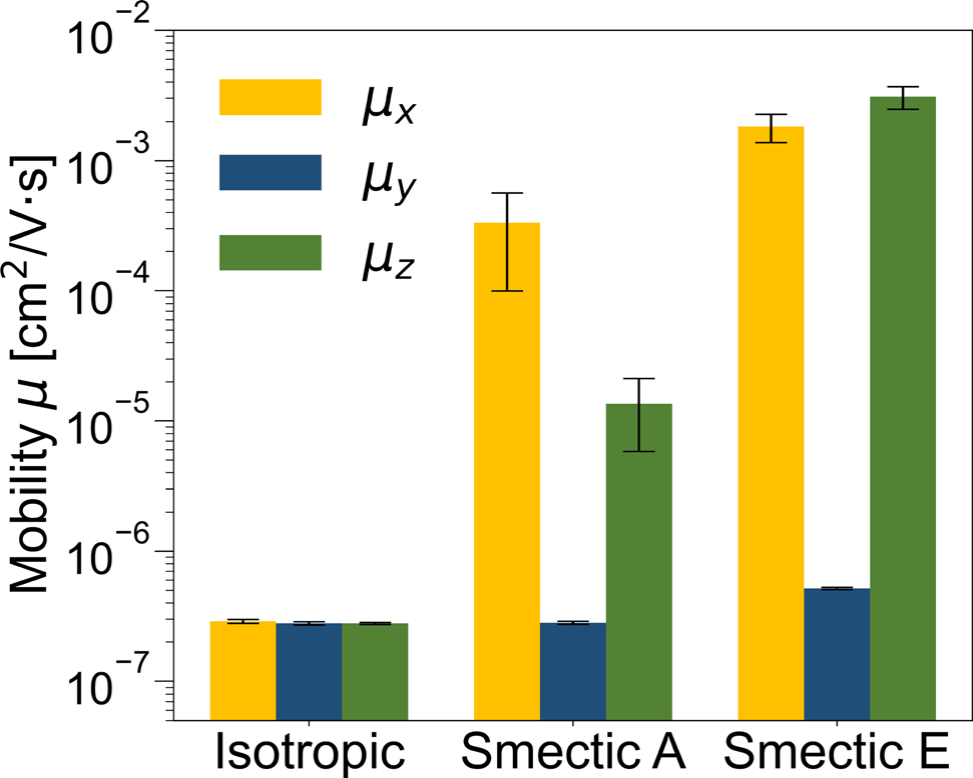
Zero-field charge mobilities along the three Cartesian coordinates (*μ*_*x*_, *μ*_*y*_, and *μ*_*z*_) for isotropic, smectic A, and smectic E phases obtained through rejection-free kMC simulations. Error bars represent the standard deviation of the mobility obtained from 100 CG configurations.

The zero-field charge mobilities along the three Cartesian coordinates (*μ*_*x*_, *μ*_*y*_, and *μ*_*z*_) for each LC phase are illustrated in Fig. 6. In the isotropic phase, the mobilities along the three coordinates exhibit similar values (<10^−6^ cm^2^ V^−1^ s^−1^) that are systematically smaller than both smectic phases due to strong disorder. Notably, the mobilities in the both smectic phases display anisotropic characteristics, with the lowest mobility observed along the *y*-direction (*μ*_*y*_ < 10^−6^ cm^2^ V^−1^ s^−1^) due to disruptions in the effective charge transport path caused by the lamellar spacing. Conversely, the *μ*_*x*_ value, approximately 10^−3^ cm^2^ V^−1^ s^−1^, can be attributed to the alignment of π–π stacking perpendicular to the LC director, as depicted in Fig. 2d. Importantly, only in the smectic E phase does *μ*_*z*_ surpass 10^−3^ cm^2^ V^−1^ s^−1^ due to the presence of a herringbone-like structure.

The isotropic mobilities (averaged over *μ*_*x*_, *μ*_*y*_, and *μ*_*z*_) for the isotropic, smectic A, and smectic E phases are ∼10^−7^, ∼10^−4^, and ∼10^−3^ cm^2^ V^−1^ s^−1^, respectively. Given that zero-field charge mobility is generally 10^3^ to 10^4^ times smaller than the field-effect mobility,^147–149^ the isotropic mobility obtained from our kMC simulation aligns well with the mobility trend and order of magnitudes observed for the benzothienobenzothiophene family in distinct LC phases through experimental measurements.^45,56^ These results underscore the robustness of our ECG approach in elucidating the relationship between morphology and electronic properties at CG resolution in a more efficient manner compared to typical multiscale approaches.

### 3.5 Examining the potential for bottom-up electronic coarse-graining over fields

Provided the scalable electronic structure predictions enabled by use of a particle-based CG model for the LC material BTBT, we assess the potential for moving into field-based CG models of electronic structure parameterized from the “bottom-up” to further extend accessible spatiotemporal simulation scales. Such field-based models are the workhorses of the Chemical Engineering and Materials Science communities, with recent work being performed to introduce systematic “bottom-up” CG approaches for structural and thermodynamic predictions.^34^ Notably, simulations of LC materials have historically been the domain of such field-based descriptions,^150,151^ motivating the assessment of the question within the specific context of LC materials.

**Fig. 7:**
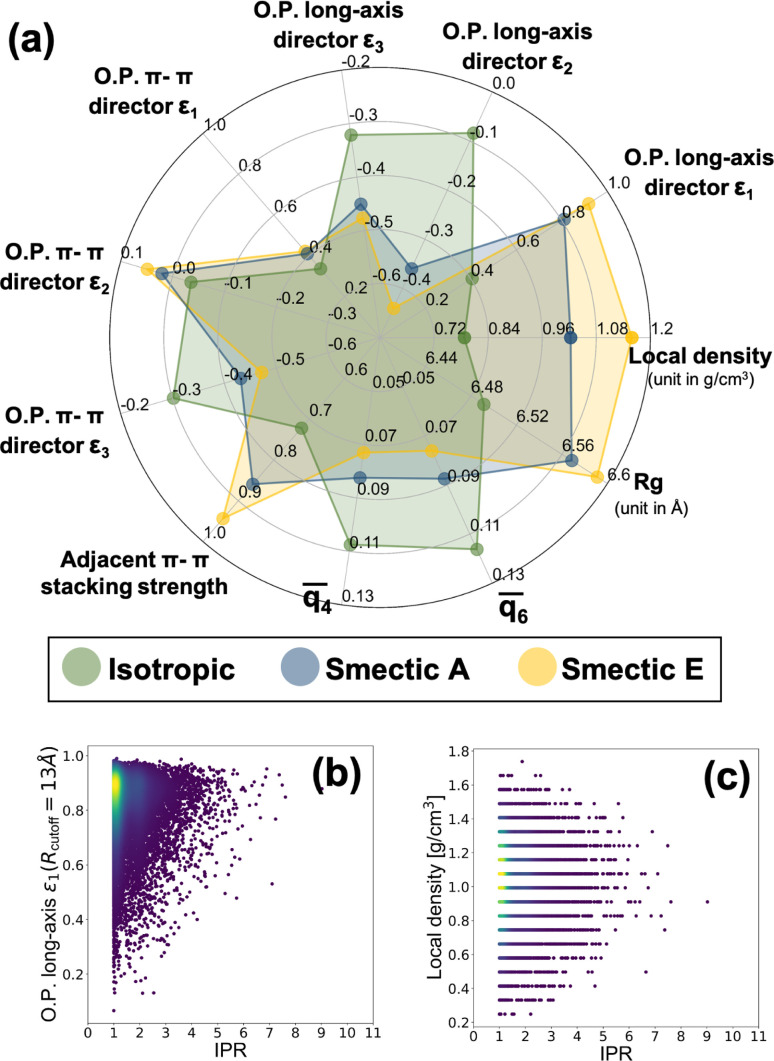
(a) Radar charts illustrating the mean values of field-based descriptors in isotropic and smectic phases, where the descriptors include O. P. long-axis *ε*_*i*_, representing the *i*_th_ eigenvalue of the nematic order tensor aligned with the BTBT long axis; O. P. π–π director *ε*_*i*_, signifying the eigenvalue based on the π–π direction; *q̄*_4_ and *q̄*_6_, denoting the 4-fold and 6-fold Steinhardt order parameters, respectively; and *R*_g_, corresponding to the radius of gyration of BTBT molecules, and (b) correlation between local nematic order parameter (O. P. long-axis *ε*_1_) and IPR, (c) correlation between local density and IPR.

To explore this, we computed a broad array of local order parameters within a characteristic radius of 13 Å, including nematic tensor order parameters of BTBT's long and π-system axes, multiple Steinhardt order parameters,^152–154^ the local density, and the local π–π-stacking strength. Fig. 7 a shows the average values of all computed field-based local order parameters for the isotropic and smectic phases, respectively, and the distributions of all field-based parameters as a function of IPR value are demonstrated in Fig. S19–S21.† Clearly, each phase can be distinguished by multiple different types of order parameters when coarse-grained from the “bottom-up.” To probe the feasibility of field-based CG descriptions of electronic structure, we assembled a data set of IPR calculated from the extracted local environments within the CG simulations of Fig. 7. As the local density and nematic order parameter are two common field-based descriptors used in soft materials theory, we analyzed whether these descriptors possessed any correlation with the resulting local electronic structure of BTBT in either LC phase. A weak visual correlation between the nematic order parameter of the long axis and the IPR was observed (Fig. 7b) but quantitative regression analysis using LASSO regression for feature selection elicited no meaningful predictive relationships. No visual correlation was observed between the local density and the ECG-computed IPR (Fig. 7c). LASSO regression and visual examination of all computed structural metrics further showed little correlation with the resulting IPR (see Fig. S19–S21 in the ESI†). This result suggests that while particle-based CG representations can be productive in scaling up electronic predictions to the mesoscale for disordered materials, field-based descriptors for the electronic structure of molecular LC are untenable at present.

Such a negative result should not be taken as evidence of a complete lack of potential for such models, but is sensible provided the standard densities and weak intermolecular couplings between LC molecules in this study. In more strongly coupled systems (higher densities, polymer chains) field-based descriptors of local electronic structure may be more predictive. Notably, in the context of semi-crystalline systems including grain boundaries and crystallites, a field-based descriptor would likely be a fruitful characterization of the local electronic structure.

## 4 Conclusions

In this work, we have integrated bottom-up CG and ECG techniques to provide the first quantitative characterization of the morphology-dependence of electronic structure in a LC semiconductor at the ∼20 nm length scale. Importantly, this framework provided such characterization with minimal computational resources and without the need for *ad nauseam* QC or complicated backmapping protocols, which enabled robust statistical analysis averaging over the full thermodynamic ensemble. This investigation revealed increased wavefunction delocalization in both smectic phases relative to the isotropic phase, as well as a recurrent two-site charge carrier motif common to the LC semiconductor BTBT. Using the CG electronic Hamiltonian, the zero-field mobility obtained *via* kMC simulations agrees semi-quantitatively with experimental mobility trends, validating the mesoscale electronic structure predictions from ECG. Importantly, we analyzed the potential for field-based ECG methods moving forward and concluded that significant work remains to be done to connect bottom-up electronic structure predictions with the mesoscopic scales that dictate soft materials function. This work marks a significant advancement in the ability to quantitatively model the relationships between multiscale morphology and electronic structure in organic semiconductors.

## Data availability

The data that support the findings of this study are available from the corresponding author upon reasonable request.

## Author contributions

C. I. Wang: methodology, software, validation, formal analysis, investigation, writing – original draft, visualization, J. C. Maier: writing – methodology, review and editing, N. E. Jackson: conceptualization, writing – review and editing, resources, supervision, project administration, funding acquisition.

## Conflicts of interest

There are no conflicts to declare.

## Supplementary Material

SC-015-D3SC06749A-s001
