# Supplementary material for: Accessing the electronic structure of liquid crystalline semiconductors with bottom-up electronic coarse-graining
Source: Chem Sci. 2024 May 2;15(22):8390–403. doi: 10.1039/d3sc06749a (PMC11151863; doi:10.1039/d3sc06749a)
Supplement: SC-015-D3SC06749A-s001 [file SC-015-D3SC06749A-s001.pdf]

Electronic Supplementary Material (ESI) for Chemical Science.
This journal is © The Royal Society of Chemistry 2024

Supporting Information: Accessing the Electronic Structure of Liquid Crystalline Semiconductors with Bottom-Up Electronic Coarse-Graining

Chun-I Wang, J. Charlie Maier, and Nicholas E. Jackson*

Department of Chemistry, University of Illinois at Urbana-Champaign, 505 S Mathews Avenue, Urbana, Illinois, 61801, USA

E-mail: jacksonn@illinois.edu

Contents

S1 Electronic Coarse-Graining (ECG) with Deep Kernel Learning (DKL) S3

S2 Details of Establishing Bottom-Up CG Models S3

S2.1 All-Atom MD Simulations for Reference CG Model S3

S2.2 CG Representation and CG Bonded Potentials S4

S2.3 Iterative Boltzmann Inversion for Non-Bonded Potentials S6

S2.4 Evaluation of Bottom-Up CG Models S8

S3 AA MD Simulation: Temperature Decrease from 700 K to 350 K S14

S4 Details of DKL-ECG for HOMO Energy Prediction S15

S5 Details of DKL-ECG for Electronic Coupling Prediction S19

S5.1 Model description S19

S5.2 Learning Couplings with DKL-ECG in a Linear Scale S20

S5.3 Learning Couplings with DKL-ECG in a Logarithmic Scale S23

S5.4 FNN Classifier Learning for the Sign of the Coupling S27

S6 Impact of Constant Onsite Energy in the Electronic Hamiltonian S29

S7 Challenges of Bulk Structural Characterization: Insights from Local Nematic Order and IPR Correlations S31

S8 Rejection-Free Kinetic Monte Carlo Algorithm for Charge Transpor Simulation S33

S9 Correlation between IPR and Field-Based Descriptors S34

References S39

S1 Electronic Coarse-Graining (ECG) with Deep Kernel Learning (DKL)

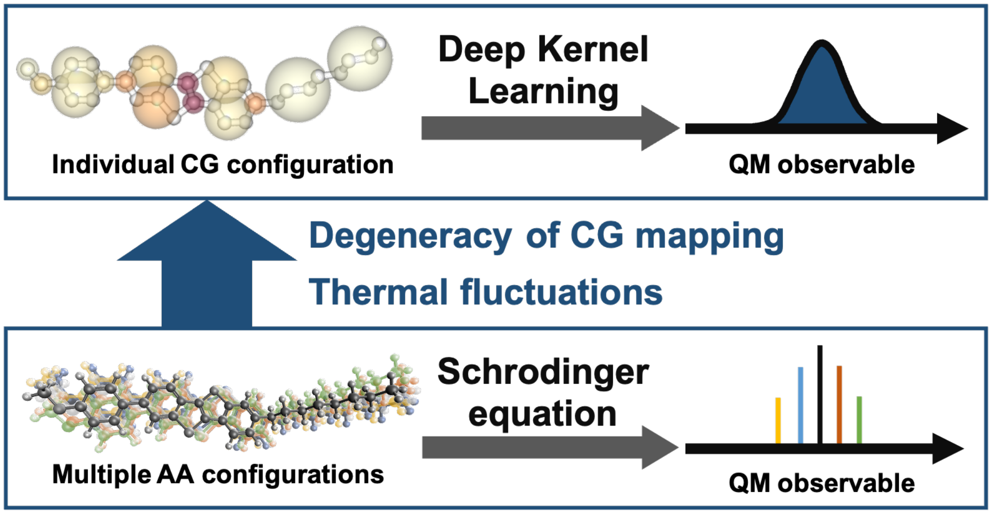

Figure S1: Schematic diagram of the DKL-ECG method: (top panel) mapping of targeted electronic property distributions (derived from all-atom (AA) configuration space and quantum chemical calculations) directly onto coarse-grained (CG) configuration space, and (bottom panel) the 'one-to-many' mapping relationship between CG and AA representations, resulting in electronic property distributions with inherent noise due to CG mapping degeneracy and thermal fluctuations.

S2 Details of Establishing Bottom-Up CG Models

S2.1 All-Atom MD Simulations for Reference CG Model

To generate the morphology of 2-(4-methoxyphenyl)-7-octyl-benzothienobenzothiophene (BTBT) in the isotropic, smectic A, and smectic E phases, which serve as reference structures for the bottom-up CG models, we conducted a series of atomistic molecular dynamics (MD) simulations. The molecular interactions in BTBT were modeled using the OPLS-based force field[S1] with reparameterization of equilibrium structure, torsional potentials, and atomic partial charges. Details regarding the parameterization of the AA force field can be found

in our previous work.[S2]

For the isotropic morphology, we initiated simulations with a system comprising 1,125 BTBT molecules within a cubic box. The system was equilibrated for 120 ns at 700 K and 1.0 bar using the Nose-Hoover NPT ensemble. A coupling constant of 1.0 ps was employed for both the thermostat and barostat. Subsequently, an additional 10 ns simulation was conducted at the same temperature under the NVT ensemble, with trajectory recordings at intervals of 20 ps. The equations of motion were integrated using the velocity Verlet algorithm with a time step of 2 fs, and hydrogen atom motion was constrained using the Rattle algorithm. Periodic boundary conditions were enforced in all three dimensions. Long-range electrostatic interactions were calculated using the particle-particle particle-mesh Ewald technique with a precision of 0.0001, and a real space cutoff of 12 Å was implemented. The same cutoff distance was utilized to truncate Lennard-Jones interactions, and geometric mixing rules were applied to different atom pairs.

For the smectic A phase, initial configurations were extracted from the final snapshot of the isotropic simulation. Equilibration and trajectory collection procedures were analogous to those used for the isotropic simulation, albeit performed at 555 K. Similarly, the smectic E phase utilized the last snapshot of the smectic A simulation, following the same simulation protocol at 515 K. These MD simulations were performed using LAMMPS.[S3]

S2.2 CG Representation and CG Bonded Potentials

The CG representation used for BTBT, depicted in Fig. S2, is determined by exploring the electronic prediction capabilities of 107 unique CG mappings, focusing on the electronic prediction of the highest occupied molecular orbital (HOMO) energy.[S2] In this CG representation, a BTBT molecule consisting of 62 atoms is systematically mapped to 15 CG particles. Table S1 lists the all-atom indices encompassed by each CG particle. As depicted in Fig.S2 and TableS1, CG-9 and CG-12 share the same carbon atom (index-28) to maintain geometric symmetry without increasing the CG resolution.

The CG bonded potentials, comprising 20 CG bond (two-body) potentials, 37 CG angle (three-body) potentials, and 58 CG torsional (four-body) potentials, were determined through direct Boltzmann inversion using the reference AA isotropic morphology. The reference probability distribution for these intramolecular CG interactions was derived by projecting the AA isotropic MD trajectory into the CG representation. We noticed that the majority of CG bond and CG angle potentials obtained through Boltzmann inversion using the reference AA isotropic morphology align well with harmonic potentials and are parametrized accordingly. However, two CG bond potentials and five CG angle potentials related to the alkyl chain manifest non-harmonic features, necessitating the application of tabulated potentials. All CG torsional potentials are effectively described by the OPLS dihedral formula. The supporting materials provide details on the tabulated potentials and fitting parameters for the harmonic CG bond, harmonic CG angle potentials, and CG dihedral potentials.

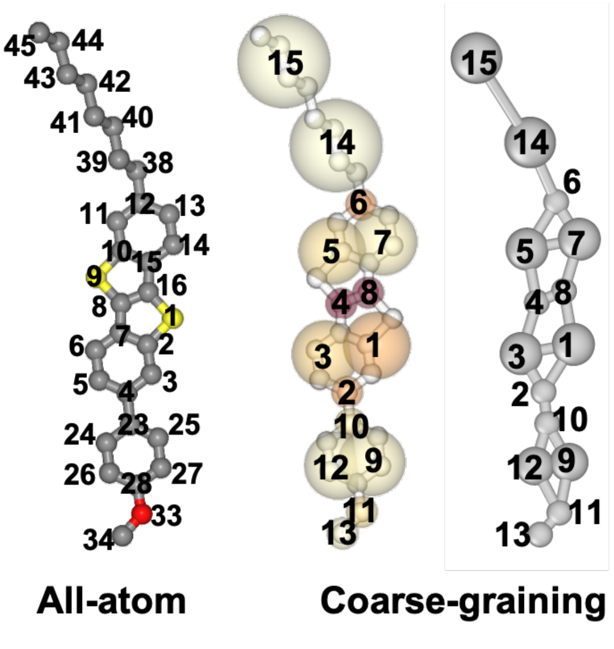

Figure S2: Molecular structure of BTBT presented in (left) all-atom representation, (middle) CG representation, and (right) the same CG representation with bonds shown. In the all-atom structure, oxygen, sulfur, and carbon atoms are denoted in red, yellow and grey, respectively, while hydrogen atoms are omitted for clarity. The all-atom indices included within each CG particle are detailed in Table S1

Table S1: All-atom indices encompassed by each CG particle

CG Index	All-atom Index
1	1, 2, 3
2	4
3	5, 6, 7
4	8
5	9, 10, 11
6	12
7	13, 14, 15
8	16
9*	24, 26, 28
10	23
11	33
12*	25, 27, 28
13	34
14	38, 39, 40, 41
15	42, 43, 44, 45

S2.3 Iterative Boltzmann Inversion for Non-Bonded Potentials

CG non-bonded pairwise potentials are derived using the Iterative Boltzmann Inversion (IBI) method[S4,S5] with pressure correction,[S6] following the equation:

$$U(r)^{(k+1)} = U(r)^k + \alpha \ln\left[\frac{g(r)^k}{g(r)^{AA}}\right] + \omega\left[1 - \frac{r}{r_{\text{cut}}}\right], \tag{S1}$$

where $U(r)^k$ denotes the CG non-bonded potential at the k_{th} iteration, $g(r)^k$ represents the radial distribution function (RDF) obtained with the k_{th} CG potential, $g(r)^{\text{AA}}$ is the reference RDF obtained from all-atom (AA) molecular dynamics (MD) simulation, α is a hyperparameter ranging from 0.01 to 0.5 for tempering the magnitude of the potential update, and ω is another hyperparameter ranging from 0 to 0.1 for scaling the pressure correction with the cutoff distance (r_{cut}) equaling 17 $\mathring{A}$. In this study, each CG particle was treated as a distinct type, resulting in the need to tabulate 120 CG non-bonded potentials using the IBI approach. Given the limited thermodynamic transferability between isotropic and LC phases, two sets of CG non-bonded potentials were established based on reference

RDFs obtained from AA MD simulations for the isotropic (700 K and 1 bar) and smectic A phases (555 K and 1 bar), respectively. For the smectic E phase (at 515 K and 1 bar), the CG non-bonded potentials were directly adopted from those designed for the smectic A phase due to their excellent transferability in reproducing the 120 RDFs, structure factor analysis and order parameter, as depicted in Fig. S4. For a comprehensive understanding of the theoretical principles of IBI, readers are directed to recent review papers.[S7–S9] Note that IBI was selected as the bottom-up coarse-graining method, as opposed to e.g. Force-Matching or Relative Entropy, due to the guarantee of structure matching as the on target parameterization property - this feature is essential for accurate subsequent prediction of the electronic structure at the CG resolution.

The CG simulations were conducted in the NVT ensemble with a Langevin thermostat damping parameter of 10 fs. Periodic boundary conditions were applied in all three dimensions. The initial configuration was derived by projecting the last snapshot of the reference AA MD trajectory to a CG representation, which involved 1125 BTBT molecules. In each iteration, the system underwent equilibration for 10,000,000 steps, followed by an additional 10,000,000 steps to estimate the $g(r)^k$. A time step of 2 fs was employed, but it was adjusted to 1 fs if the CG potentials led to an unstable simulation. Notably, the strongly anisotropic molecular interactions combined with BTBT's rigid fused ring motifs, the isotropically floppy alkyl side chain, and a highly asymmetric structure posed substantial challenges to the IBI process in the context of smectic phases. For instance, the conventional approach of initializing CG potentials through Boltzmann inversion of RDFs derived from the AA MD reference, a standard starting point for IBI, resulted in the divergence of the IBI process. To address this challenge, we adopted isotropic CG non-bonded potentials as an alternative initial guess for the IBI procedure. However, even with this modification, achieving convergence for the smectic A CG model required over 70 iterations to meet the convergence criterion (average RDF error smaller than 0.01), while the isotropic CG model achieved this criterion in approximately 40 iterations. A more challenging scenario emerged when we tried to establish

individual CG non-bonded potentials for the smectic E phase. Despite using the smectic A CG non-bonded potentials as initial guesses, we encountered frequent occurrences of particle overlap, resulting in system instability and explosion. Even adjusting the value of α to 0.01 in Eq. S1 or employing a smaller time step of 0.1 fs did not prevent the unstable conditions during the IBI process for smectic E. Fortunately, the CG non-bonded potentials derived for the smecticA phase already demonstrated excellent transferability to the smecticE phase. We hypothesize that the update steps could be made more effectively by integrating the anisotropic of the nematic director into the update criterion, but such efforts are beyond the scope of the present work.

S2.4 Evaluation of Bottom-Up CG Models

To assess the accuracy of the isotropic and smectic-A bottom-up CG models, as well as to examine the transferability of the smectic-A model to the smectic-E phase, we conducted thorough structural analyses by comparing AA and CG simulations. This comprehensive evaluation included the RDFs of the center of mass (COM) (Fig. S3a), nematic order parameters (Fig.S3b), structure factor analyses (Fig.S3c-e), and RDFs of the 120 distinctive CG pairs (Figs.S4-S7). The RDFs of the COM and the 120 distinctive CG pairs exhibited excellent agreement between AA and CG simulations in isotropic and smectic phases. This agreement suggests that the CG models effectively capture short-range structural features. Moreover, structure factor analyses revealed the accurate representation of $\pi - \pi$ stacking ($q = 0.21$ Å^{-1}) and layered structure ($q = 0.03$ Å^{-1}) in both smectic phases. This observation indicates the good transferability of the smectic A bottom-up CG model to the smectic E phase and the capability to capture the long-range structural features. In summary, our CG models successfully reproduce both short-range and long-range structural features.

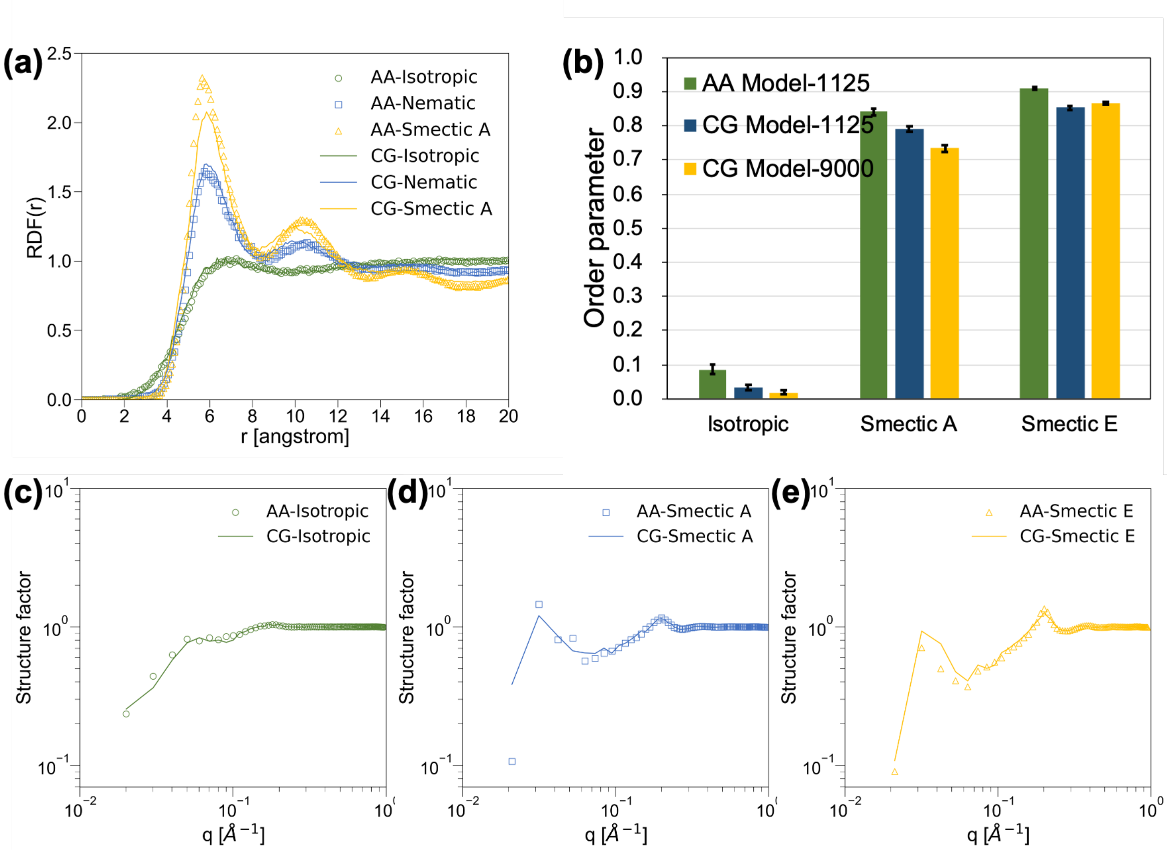

Figure S3: Structural analyses comparing AA and CG models in isotropic, smectic A, and smectic E phases: (a) RDFs of the COM, (b) nematic order parameters, where "CG Model-1125" denotes results from the IBI process, while "CG Model-9000" corresponds to CG simulations with 9,000 BTBT molecules, and (c-e) structure factor analyses for isotropic, smectic A, and smectic E phases with a system of 1,125 BTBT molecules.

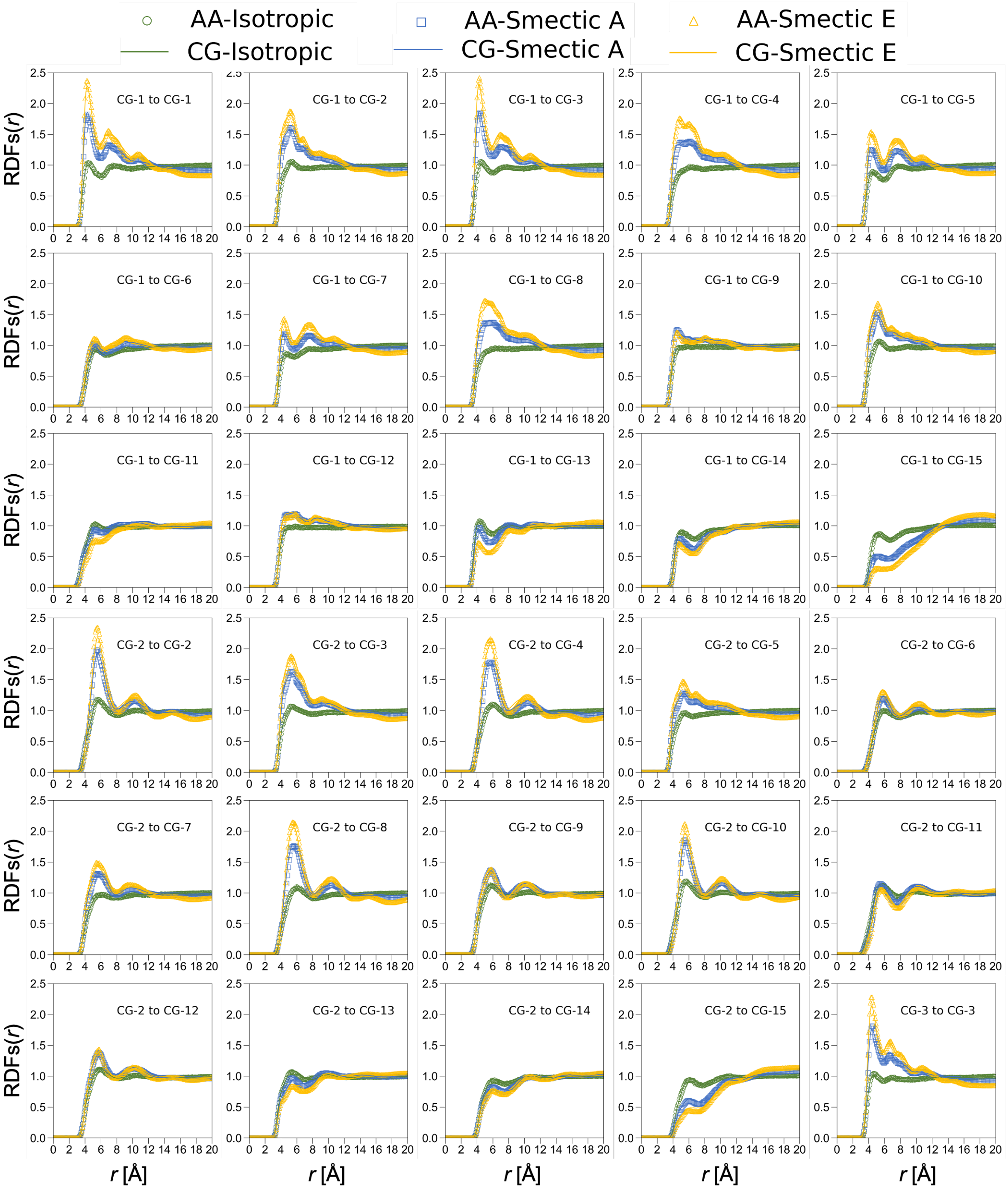

(Continued on the next page)

Figure S4: Thirty RDFs of the 120 distinctive CG represented pairs obtained from AA simulations (marked symbols) and CG simulations (solid lines) in the isotropic phase (green), smectic A phase (blue), and smectic E phase (yellow).

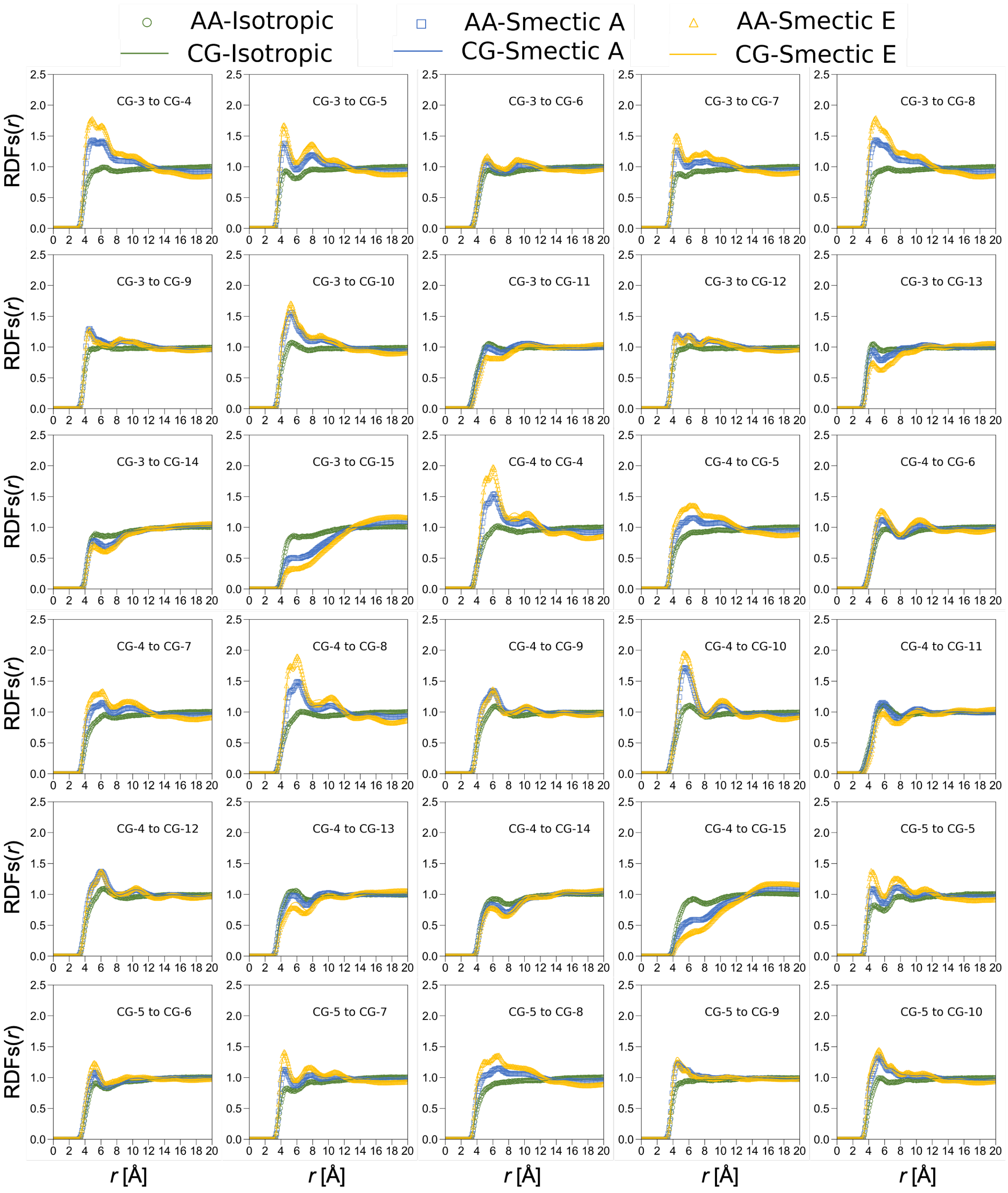

(Continued on the next page)

Figure S5: Another thirty RDFs of the 120 distinctive CG represented pairs obtained from AA simulations (marked symbols) and CG simulations (solid lines) in the isotropic phase (green), smectic A phase (blue), and smectic E phase (yellow).

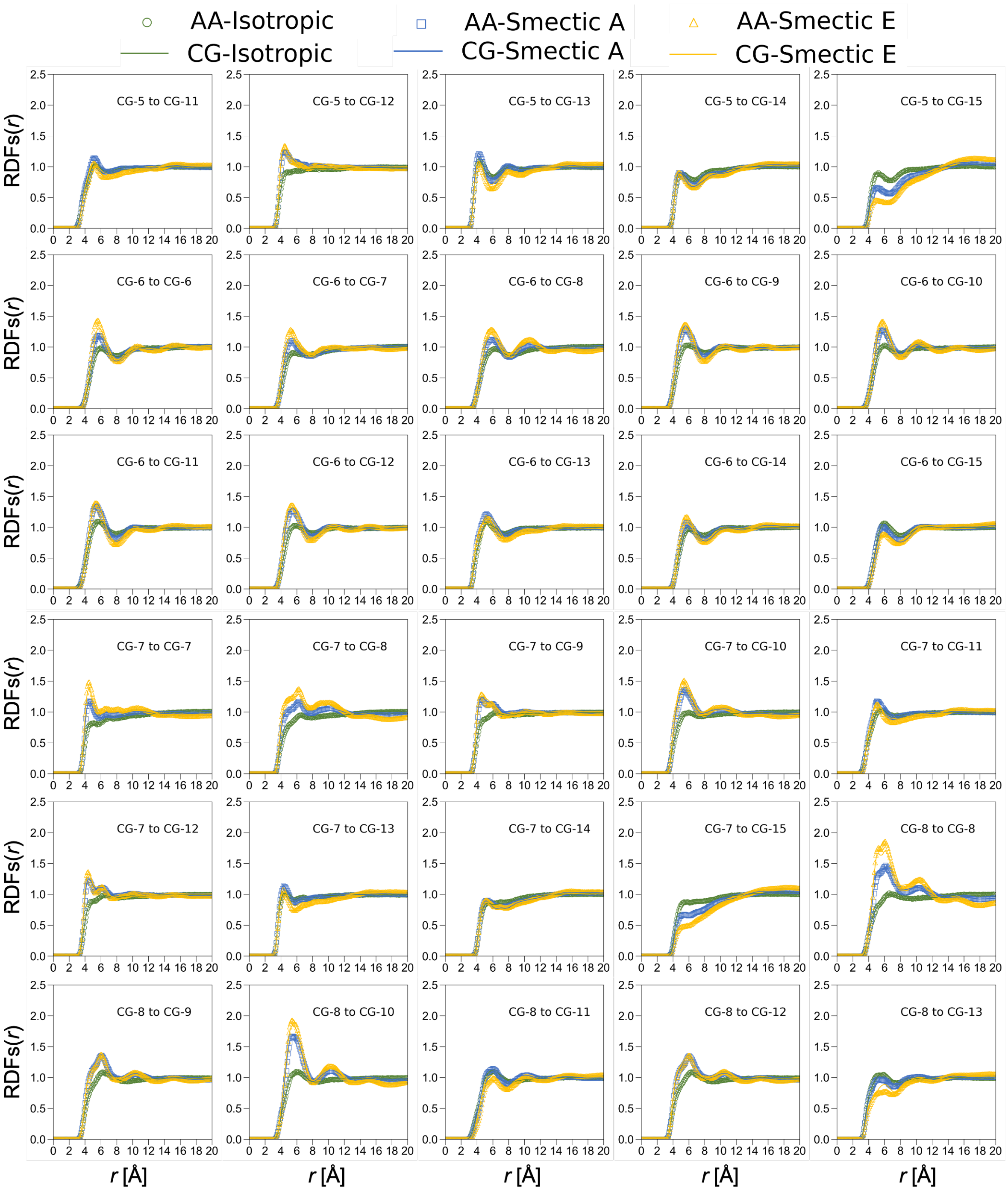

(Continued on the next page)

Figure S6: Another thirty RDFs of the 120 distinctive CG represented pairs obtained from AA simulations (marked symbols) and CG simulations (solid lines) in the isotropic phase (green), smectic A phase (blue), and smectic E phase (yellow).

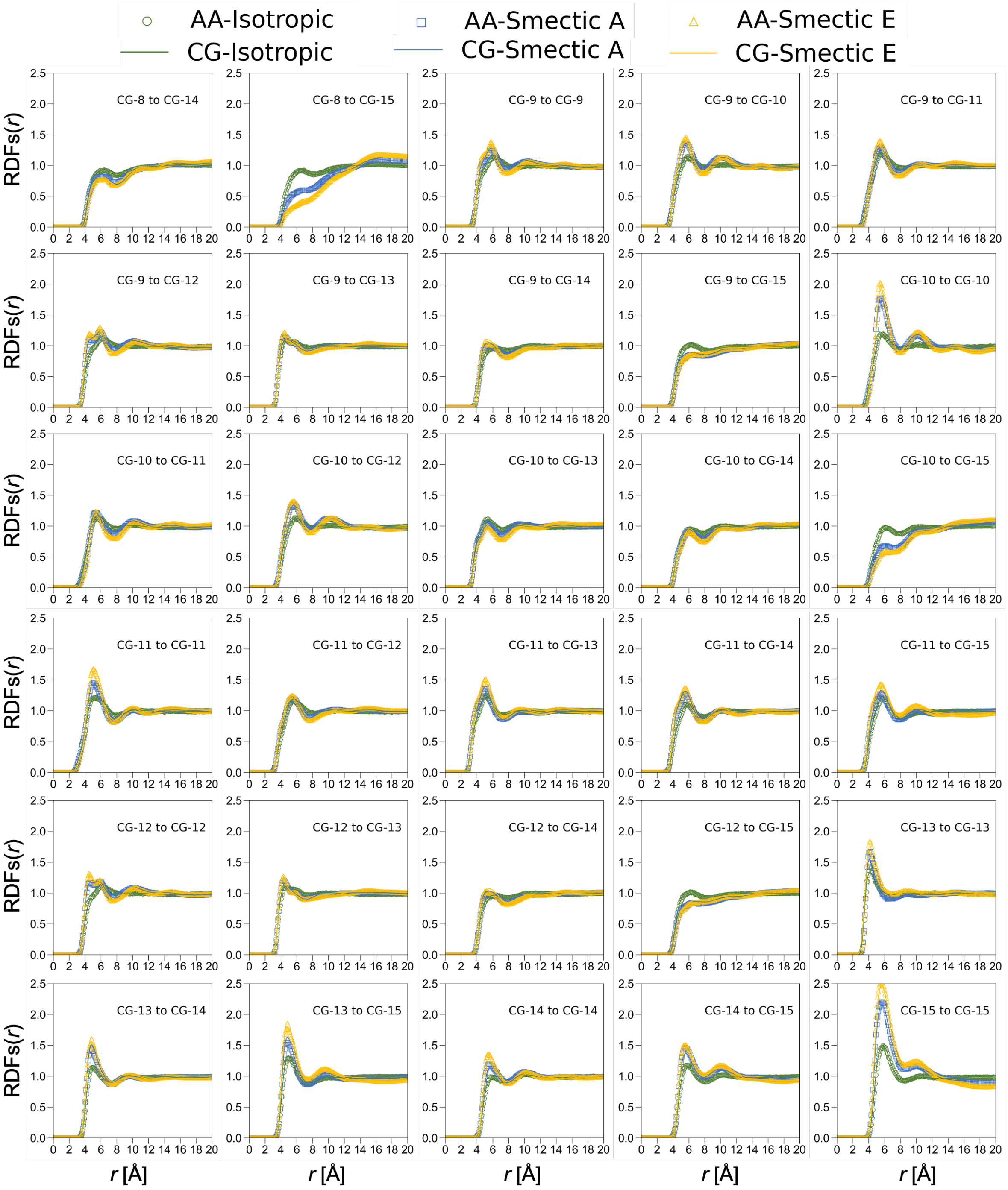

Figure S7: Another thirty RDFs of the 120 distinctive CG represented pairs obtained from AA simulations (marked symbols) and CG simulations (solid lines) in the isotropic phase (green), smectic A phase (blue), and smectic E phase (yellow).

S3 AA MD Simulation: Temperature Decrease from 700 K to 350 K

We conducted an additional AA MD simulation wherein the system temperature linearly decreased at a rate of 3.5 K/ns from 700 K to 350 K. The initial configuration for this simulation with 1,125 BTBT molecules was extracted from the last snapshot of the 700K trajectory discussed in the previous section. The simulation parameters remained consistent with those used in the previous AA MD simulations, which served as the reference for the bottom-up CG models. Figure S8 illustrates the distribution of the structure factor as well as the nematic order parameters for the molecular long-axis and short-axis, with respect to decreasing temperatures.

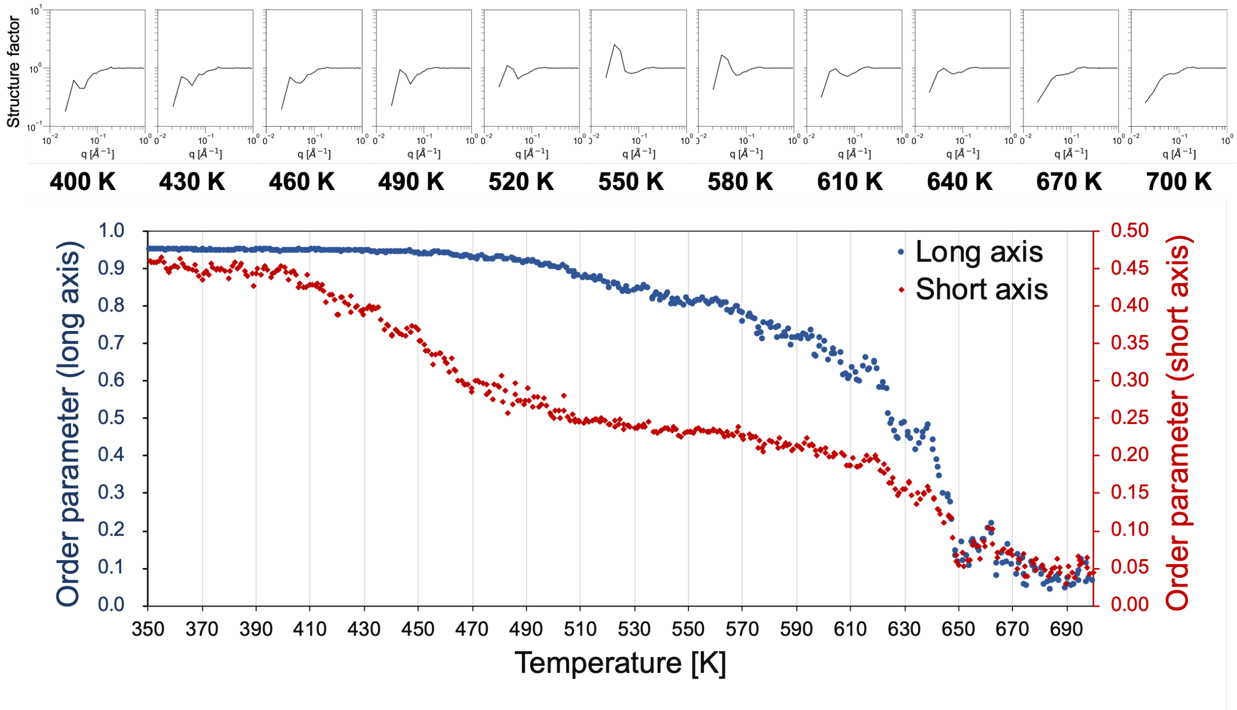

Figure S8: Structural analyses in AA MD simulation across decreasing temperatures (700 K to 350 K): structure factor distribution (top panels) and nematic order parameters for the molecular long-axis and short-axis (bottom panels).

S4 Details of DKL-ECG for HOMO Energy Prediction

In this study, we conducted MD simulations at an atomistic resolution to generate a diverse set of BTBT monomer configurations in the isotropic and both smectic phases. The isotropic and smectic data sets each encompass 130,000 monomer structures for training, and 10,240 monomer structures for testing. These AA monomer structures were subsequently projected onto CG representations and featurized using the inverse distance matrix as the input feature for the DKL-ECG models. To train and validate the models, the HOMO energy of each monomer configuration was computed using DFT calculations at the ωB97XD/cc-pVDZ level, serving as the ground truth labels. Further details regarding the construction of the data set can be found in our previous work.[S2]

The DKL-ECG model comprises a feedforward neural network (FNN), a variational layer, upon which Gaussian process regression (GPR) is performed. Model optimization involved tuning the FNN architecture, the latent space size of the variational layer, initial learning rate, and batch size. Details of these hyperparameters and other model settings are provided in Table S2. Interestingly, we observed that model performance showed minimal sensitivity to increased neural network complexity. Attempts to enhance accuracy by augmenting the FNN architecture with more hidden layers and nodes or expanding the latent space of the variational layer did not yield substantial benefits (data not shown). Notably, our prior investigations have emphasized the pivotal role of the CG representation in influencing the ECG model's accuracy in predicting HOMO energy. Specifically, for BTBT, a precise preservation of atomic coordinates in the CG representation, capturing higher electron density at the HOMO level, significantly influenced model performance.[S2]

As depicted in Fig. S9 a-c and Fig. S10 a-c, the ensemble averages from the ECG models consistently aligned with the distributions of both training and testing data sets.In Fig. S9 d-f and Fig. S10 d-f, the R^2 values between DFT calculations and ECG-predicted means consistently hovered around 0.7 with the mean absolute error (MAE) around 0.06 eV. Fig. S9

Table S2: Choice of DKL-ECG hyperparameters for HOMO energy prediction.

Hyperparameter name	Value
FNN architecture[a]	(80, 40, 20, 10)*<br>(200, 100, 50, 20)<br>(500, 400, 300, 200, 100, 50)
Activation function	leaky ReLU
Laten space size of variation layer	6*, 15, 20
No. of inducing points of GPR	2000
Optimizer	Adam
Loss function	evidence lower bound (ELBO)
No. of epoch	1000
Initial learning rate	0.02*, 0.002<br>(reduce by a factor of 0.5 every 200 epochs)
Batch size	1000, 10000*, 15000
Cross validation	5-fold

[a] The dimensions within the parentheses indicate the total number of FNN hidden layers, with the elements specifying the number of nodes in each respective hidden layer.

* The optimized hyperparameters of the DKL-ECG model were determined through training on the isotropic dataset.

further highlights the ECG model's impressive transferability from the Testing-Isotropic data set to the Testing-Smectic A and the Testing-Smectic E data sets. This transferability can be attributed to the broader configuration space covered by the isotropic data set, as validated by a principal component analysis (PCA) conducted on both data sets shown as Fig. S11.

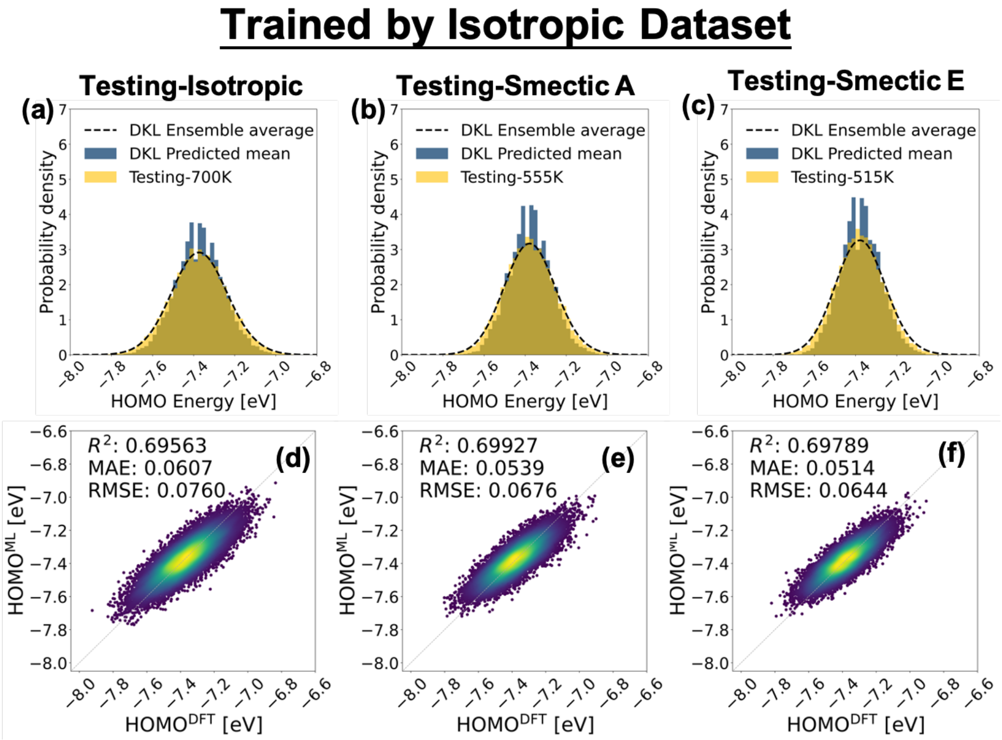

Figure S9: Performance evaluation of the DKL-ECG model for predicting HOMO energy: (Upper panels) HOMO energy distribution based on ensemble averages from ECG predictions shown as black dashed lines, HOMO density of states obtained through DFT calculations illustrated as a yellow histogram, and ECG-predicted mean values depicted in blue histograms, and (Lower panels) a comparison between HOMO energy obtained through DFT calculations and the mean values predicted by the DKL-ECG models, trained on the Training-Isotropic data set and validated using the Testing-Isotropic data set (a and d), the Testing-Smectic A (b and e) and Testing-Smectic E (c and f).

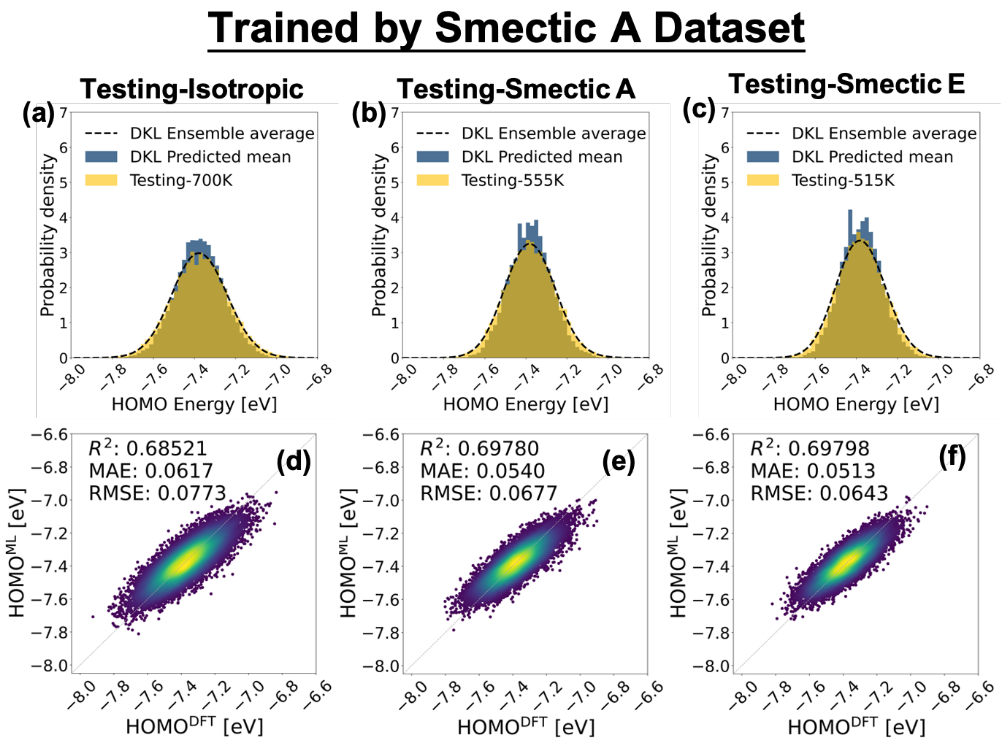

Figure S10: Performance evaluation of the DKL-ECG model for predicting HOMO energy: (Upper panels) HOMO energy distribution based on ensemble averages from ECG predictions shown as black dashed lines, HOMO density of states obtained through DFT calculations illustrated as a yellow histogram, and ECG-predicted mean values depicted in blue histograms, and (Lower panels) a comparison between HOMO energy obtained through DFT calculations and the mean values predicted by the DKL-ECG models, trained on the Training-Smectic A data set and validated using the Testing-Isotropic data set (a and d), the Testing-Smectic A (b and e) and Testing-Smectic E (c and f).

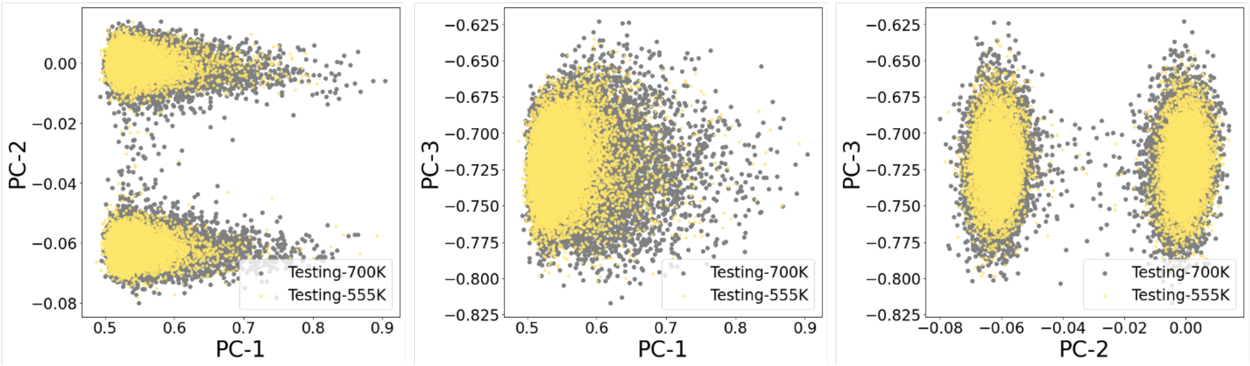

Figure S11: Principal component analysis of the first three principal components of the CG configuration space for the Testing-Isotropic data set (grey dots), where these components contribute to 50 percent of the variance. The projection of the Testing-Nematic data set is based on the principal components of the Testing-Isotropic data set (yellow dots).

S5 Details of DKL-ECG for Electronic Coupling Prediction

S5.1 Model description

The data sets for the HOMO-HOMO electronic couplings of the dimers were obtained through MD simulations at an atomistic resolution in isotropic and both smectic phases. We sampled BTBT pair structures with a separation distance of their COM less than 7 $\AA$ from the same MD trajectories used to establish the HOMO energy data set. The isotropic data set for electronic coupling comprises 125,000 molecular pairs for training and 10,240 for testing, while the smectic A data set includes 90,000 pair structures for training and 10,240 for testing. For coupling prediction, the input features were the inverse distance matrix of CG coordinates based on the sampled atomistic pair structures, but only the matrix elements obtained from intermolecular CG particles were included. Matrix elements calculated by intramolecular CG particles were excluded as they led to a reduction in model accuracy. The ground truth coupling value of each molecular pair was calculated through DFT calculations and the dimer projection technique[S10–S12] along with a phase matching approach,[S13,S14] using the sampled atomistic configurations as input. Details regarding the DKL-ECG architecture, hyperparameters, and other model settings can be found in Table S3.

Table S3: Choice of DKL-ECG hyperparameters for electronic coupling prediction.

Hyperparameter name	Value
FNN architecture[a]	(80, 40, 20, 10)<br>(200, 100, 50, 20)*<br>(500, 400, 300, 200, 100, 50)
Activation function	leaky ReLU
Laten space size of variation layer	6*, 15, 20
No. of inducing points of GPR	2000
Optimizer	Adam
Loss function	evidence lower bound (ELBO)
No. of epoch	4000
Initial learning rate	0.02*, 0.002<br>(reduce by a factor of 0.5 every 200 epochs)
Batch size	1000, 5000*, 10000
Cross validation	5-fold

[a] The dimensions within the parentheses indicate the total number of FNN hidden layers, with the elements specifying the number of nodes in each respective hidden layer.

* The optimized hyperparameters of the DKL-ECG model were determined through training on the smectic A dataset and learning coupling in a logarithmic scale.

S5.2 Learning Couplings with DKL-ECG in a Linear Scale

Machine learning for predicting electronic coupling has proven to be a challenging task, especially considering the vast variability in the alignment between pairs of molecules, leading to coupling values spanning several orders of magnitude, particularly in non-crystalline phases.[S13,S14] The conventional approach in state-of-the-art machine learning models for coupling prediction involves training with input features derived from atomic coordinates. Notably, it has been observed that models learning coupling in a linear scale generally outperform those learning in a logarithmic scale, as sharper changes in log-scale coupling values become apparent in regions where the pair configurations induce a switch in coupling sign.[S13] However, in our investigation using input features derived from CG coordinates, we observed the opposite trend. DKL-ECG models learning the primitive coupling value (linear scale with sign) performed poorly, as depicted in Fig. S12. Two key factors contribute to these results:

1) the use of CG coordinates as input features, mitigating the impact of transition regions where coupling changes sign, and 2) the application of the evidence lower bound (ELBO) loss function under the DKL framework. The ELBO function is designed to minimize the divergence between the distribution of the training dataset and the overall predictive distribution. The predictive distribution is composed of the predictive mean and predicted variance for each data point. In Fig. S12 d, the predictive distribution (depicted by the black dashed line) aligns seamlessly with the training dataset (illustrated by the yellow histogram) when the DKL model learns couplings in a linear scale. However, this DKL model faces challenges in distinguishing large-coupling pair conformations. It erroneously assigns these pair conformations the same predictive mean value of either 100 or -100 meV, resulting in the observed cut-off pattern of the coupling mean value in Fig. S12 c and d. In contrast, Fig. S12 a and b depict another extreme scenario where the DKL model predicts a similar mean value (0 meV) for all data points. Nevertheless, the presence of different predicted variances leads to a small divergence between the distribution of the training dataset and the overall predictive distribution.

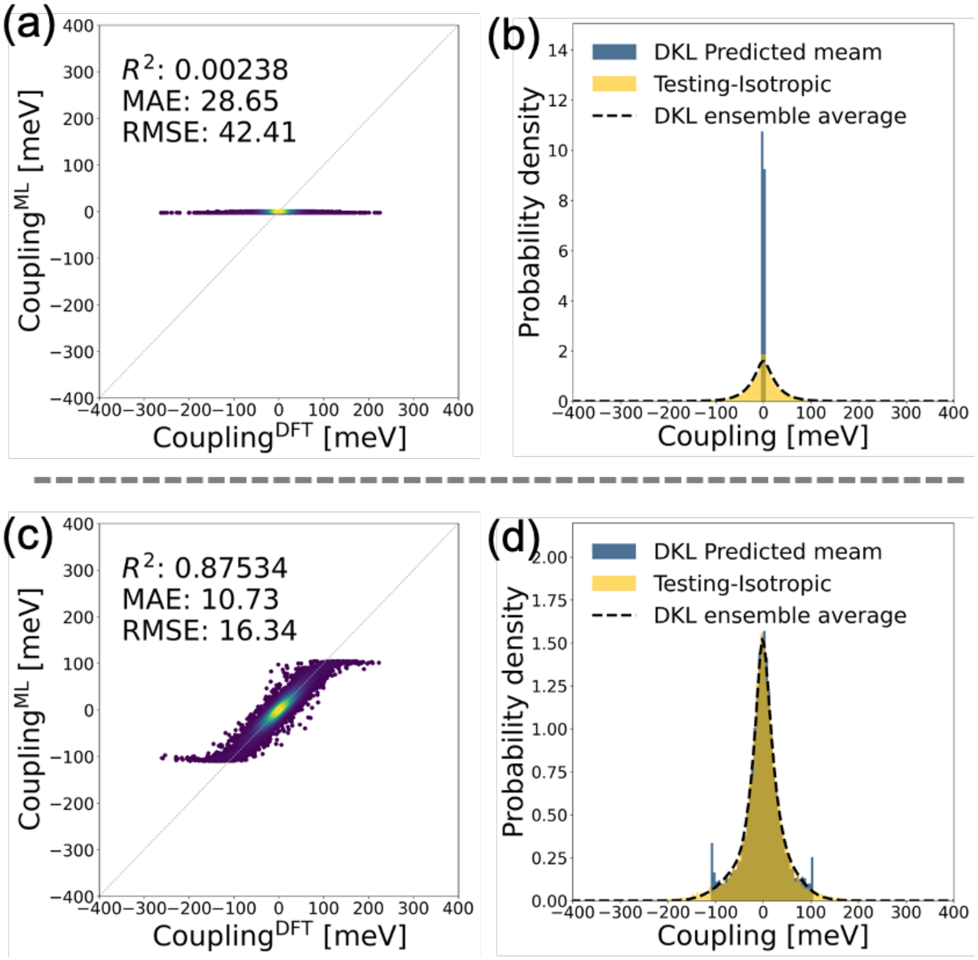

Figure S12: Performance evaluation of the DKL-ECG models for predicting HOMO-HOMO electronic coupling, trained and validated on the isotropic data set (upper panels) and the smectic A data set (lower panels): (a, c) a comparison between the HOMO-HOMO coupling distributions obtained through DFT calculations and the mean values predicted by the DKL-ECG models, and (b, d) distributions of coupling values obtained through DFT calculations (yellow histogram), distributions of ensemble averages from ECG predictions (black dashed lines), and distributions of DKL-predicted mean values (blue histograms).

S5.3 Learning Couplings with DKL-ECG in a Logarithmic Scale

Due to the poor performance of the DKL-ECG model in learning the HOMO-HOMO electronic coupling in a linear scale, we opted to train the model to predict coupling in a logarithmic scale. Following ECG predictions in a logarithmic scale, the predicted values are converted to a linear scale, and the sign of the coupling value is determined by a sign classifier introduced in the following section for the construction of the electronic Hamiltonian. Figures S13 and S14 illustrates the model's performance and transferability across isotropic and different LC phases. The DKL-ECG model trained by Smectic A dataset outperforms the one trained by Isotropic dataset. As depicted in Fig. S14 a-c, DKL models learning the logarithmic scale labels successfully predict couplings in the larger coupling (>100 meV or <-100 meV) regions, which play a significant role in the the ultimate charge transport properties of the film. In addition, while the predicted coupling for a given molecular pair is described by a Gaussian distribution, the distribution of ensemble averages still reasonably captures the data set distribution, as depicted in Fig. S14 d-f. To further enhance the predictive power of DKL-ECG, a potential avenue is conducting a transformation that converts the data set to a Gaussian distribution, a topic we aim to address in our future work.

In terms of model transferability, the general expectation is that data sets collected from isotropic morphologies cover a more comprehensive coordinate space, resulting in better transferability for other LC phases. Surprisingly, our observations reveal that the model trained on the smectic A data set exhibits exceptional transferability, with R^2 values exceeding 0.7 and an MAE around 10 meV, surpassing the performance of the model trained on the isotropic data set. PCA on both data sets, as depicted in Fig S15, indicates that the isotropic data set covers a more extensive coordinate space than the smectic A data set. Additionally, the PCA reveals two distinctive groups, corresponding to the angle between the long axes of BTBT pairs, as illustrated in Fig S15. One potential explanation for the superior transferability of the model trained on the smectic A data set is that the coupling value

depends on the complex interplay between molecular orbitals, and the diverse isotropic data set increases the difficulty of capturing the two major molecular alignments in comparison with the smectic A data set.

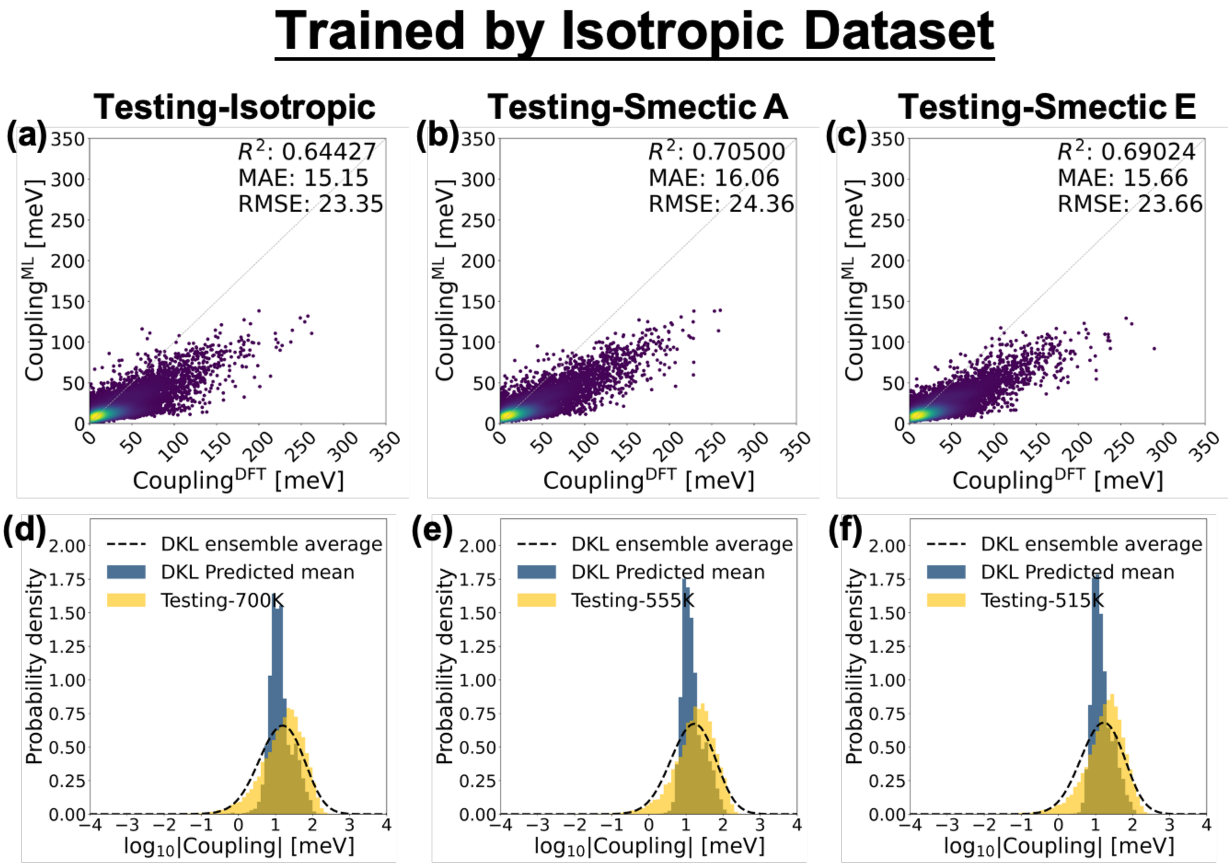

Figure S13: Evaluation of DKL-ECG Models for predicting the HOMO-HOMO electronic coupling in logarithmic scale: (Upper panels) a comparison between electronic coupling values obtained through DFT calculations and the mean values predicted by the DKL-ECG models after the conversion from the logarithmic scale to the linear scale, and (Lower panels) distributions of coupling values obtained through DFT calculations (yellow histogram), distributions of ensemble averages from ECG predictions (black dashed lines), and distributions of DKL-predicted mean values (blue histograms). The models were trained on the Training-Isotropic data set and validated using the Testing-Isotropic data set (a and d), the Testing-Smectic A (b and e) and Testing-Smectic E (c and f).

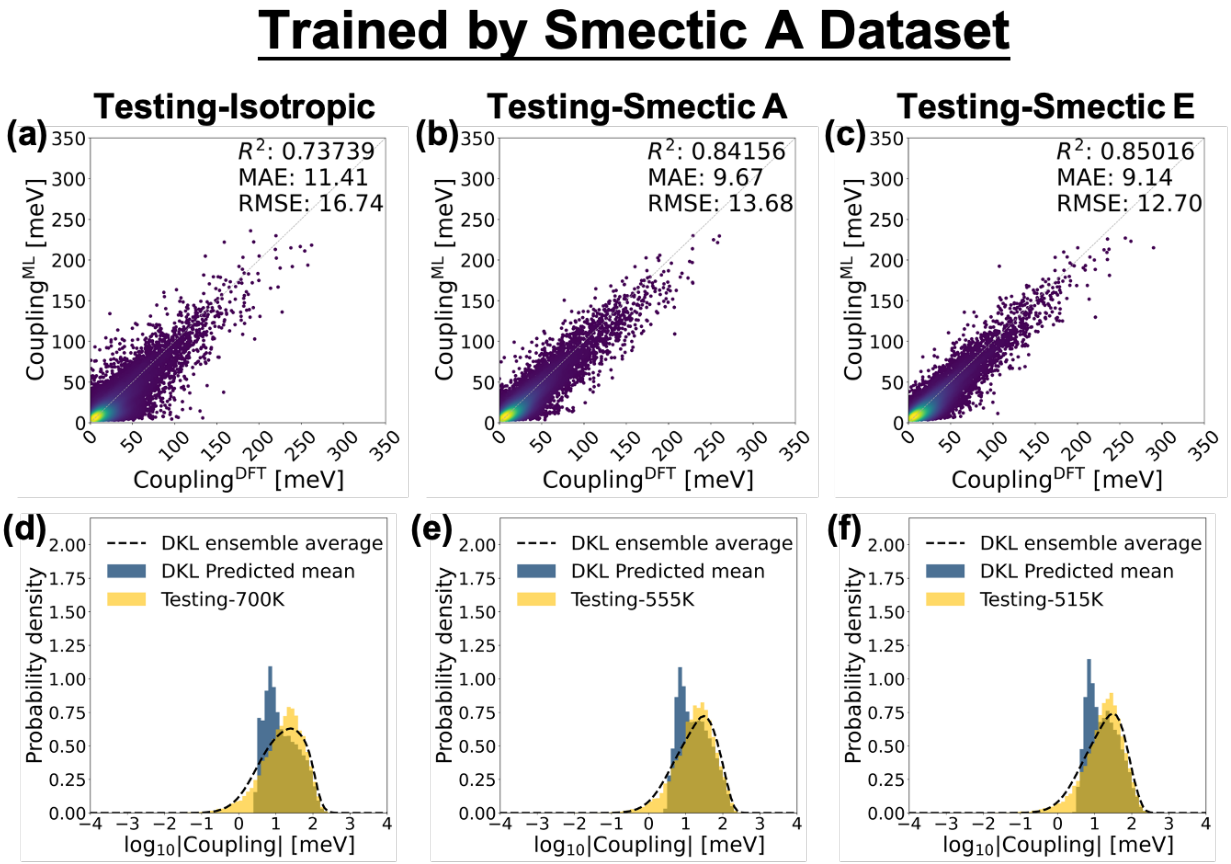

Figure S14: Evaluation of DKL-ECG Models for predicting the HOMO-HOMO electronic coupling in logarithmic scale: (Upper panels) a comparison between electronic coupling values obtained through DFT calculations and the mean values predicted by the DKL-ECG models after the conversion from the logarithmic scale to the linear scale, and (Lower panels) distributions of coupling values obtained through DFT calculations (yellow histogram), distributions of ensemble averages from ECG predictions (black dashed lines), and distributions of DKL-predicted mean values (blue histograms). The models were trained on the Training-Smectic A data set and validated using the Testing-Isotropic data set (a and d), the Testing-Smectic A (b and e) and Testing-Smectic E (c and f).

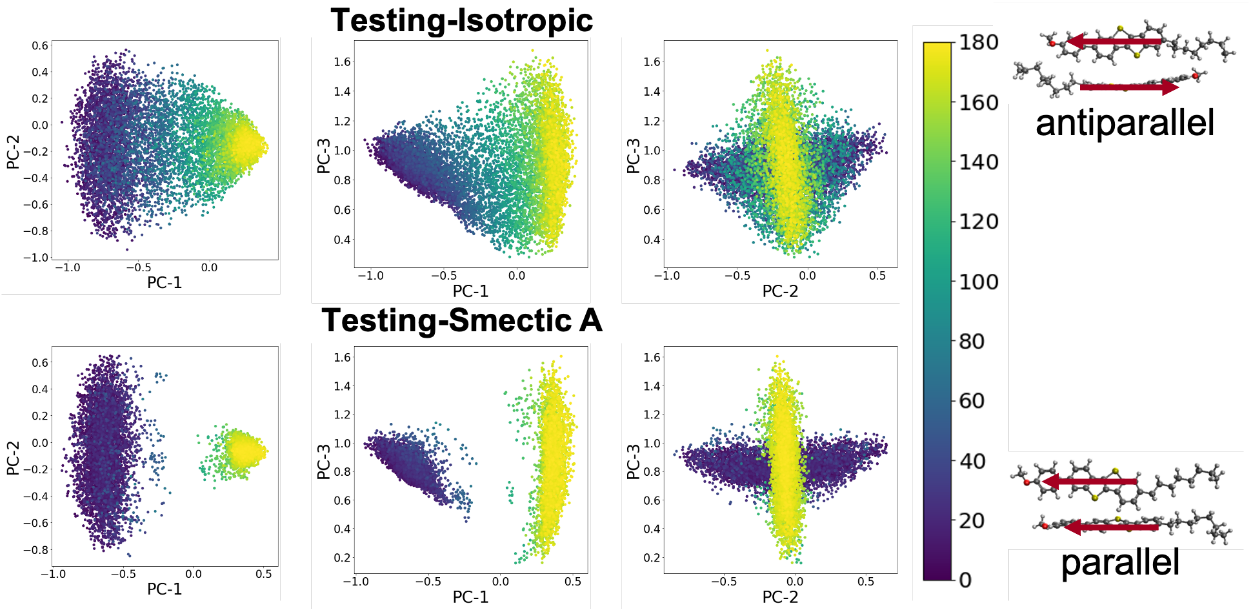

Figure S15: Principal component analysis depicting the first three principal components of the CG configurations for the (upper panels) Testing-Isotropic data set and (lower panels) Testing-Smectic A data set, with these components contributing to 72 percent and 80 percent of the variance, respectively. The heatmap illustrates the angle between the long axes of BTBT pairs.

S5.4 FNN Classifier Learning for the Sign of the Coupling

The DKL-ECG model, when learning the HOMO-HOMO electronic coupling in logarithmic scale, can only provide the absolute value of the coupling. To address this issue, we introduced a coupling sign classifier using an FNN. The data sets used to train the FNN classifier are the same as those for training coupling values. Notably, for both data sets, approximately 50 percent of the data exhibit a positive sign, ensuring a balanced data set. Specifics regarding FNN architecture, hyperparameters and other model settings for the FNN classifier can be found in Table S4. Figure S16 summarizes the model accuracy of the FNN classifiers trained on both data sets across varying model complexities. These results indicate that FNN classifiers trained on the isotropic data set demonstrate superior model transferability, achieving an accuracy exceeding 83 % in both LC phases, and the model performance remains relatively insensitive to changes in model complexity. The FNN classifier trained on the isotropic data set is integrated with the DKL-ECG for coupling prediction to define the off-diagonal elements of the electronic Hamiltonian.

Table S4: Choice of FNN hyperparameters for sign classification.

Hyperparameter name	Value
FNN architecture[a]	(200, 100, 64, 32)<br>(500, 400, 300, 200, 100, 50, 32)*
Activation function	leaky ReLU
Optimizer	Adam
Loss function	binary cross entropy
No. of epoch	500
Constant learning rate	0.1, 0.05, 0.01, 0.005*, 0.001
Batch size	500
Cross validation	5-fold

[a] The dimensions within the parentheses indicate the total number of FNN hidden layers, with the elements specifying the number of nodes in each respective hidden layer.
* The optimized hyperparameters of the FNN model were determined through training on the isotropic dataset.

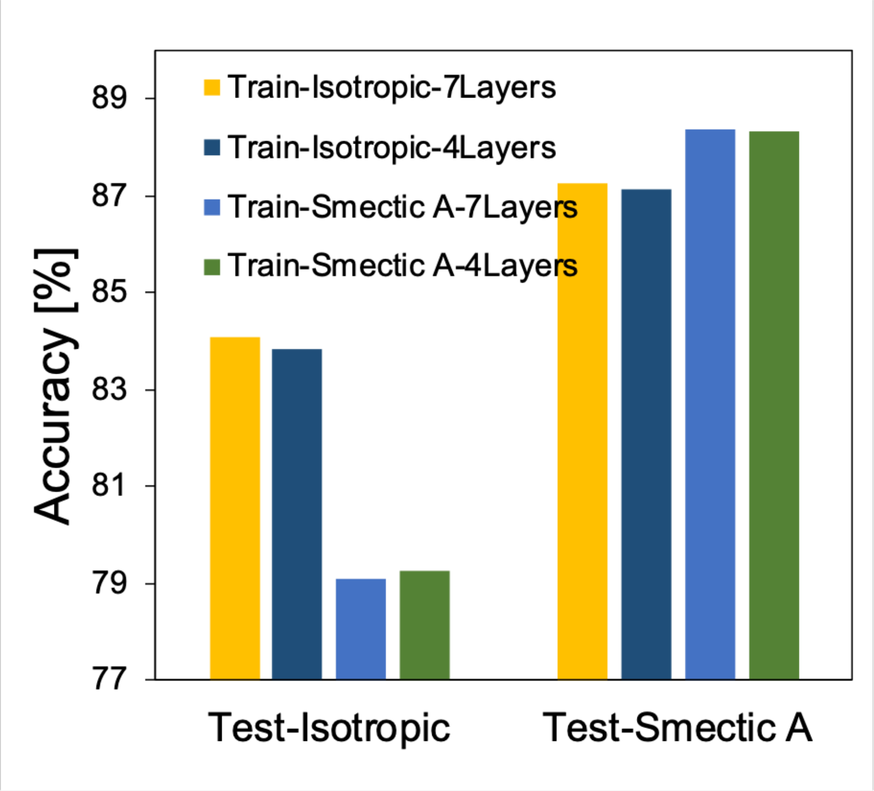

Figure S16: Model accuracy of the FNN classifiers trained on different datasets with varying model complexity.

S6 Impact of Constant Onsite Energy in the Electronic Hamiltonian

To assess the influence of onsite energy disorder, which reflects the variation in the HOMO energy (the diagonal elements of the Hamiltonian) due to the conformational disorder of BTBT, we redefined the 2,400 Hamiltonians by setting all diagonal elements to the mean value of HOMO energy across all molecules in the training data sets. Subsequently, we computed the eigenstates, eigenvectors, and inverse participation ratio (IPR) values for these modified Hamiltonians. In contrast to the case with onsite energy disorder (as shown in Fig. 4 in the main text), Fig. S17 a demonstrates that charge carriers exhibit the ability to delocalize across 10 to 3000 molecules in 9% of delocalized electronic states for the isotropic and smectic A phases and 4.3% for the smectic E phase. These findings suggest that, with constant onsite energy implying identical conformations for all BTBT molecules, a charge carrier could have highly delocalized CT states, resembling the behavior observed in other organic semiconductor systems in crystalline phases. Furthermore, comparing Fig. S17 b-d with Fig. 3 b-d in the main text, the pattern of two-molecule charge carriers remains unchanged when the onsite energy is constant.

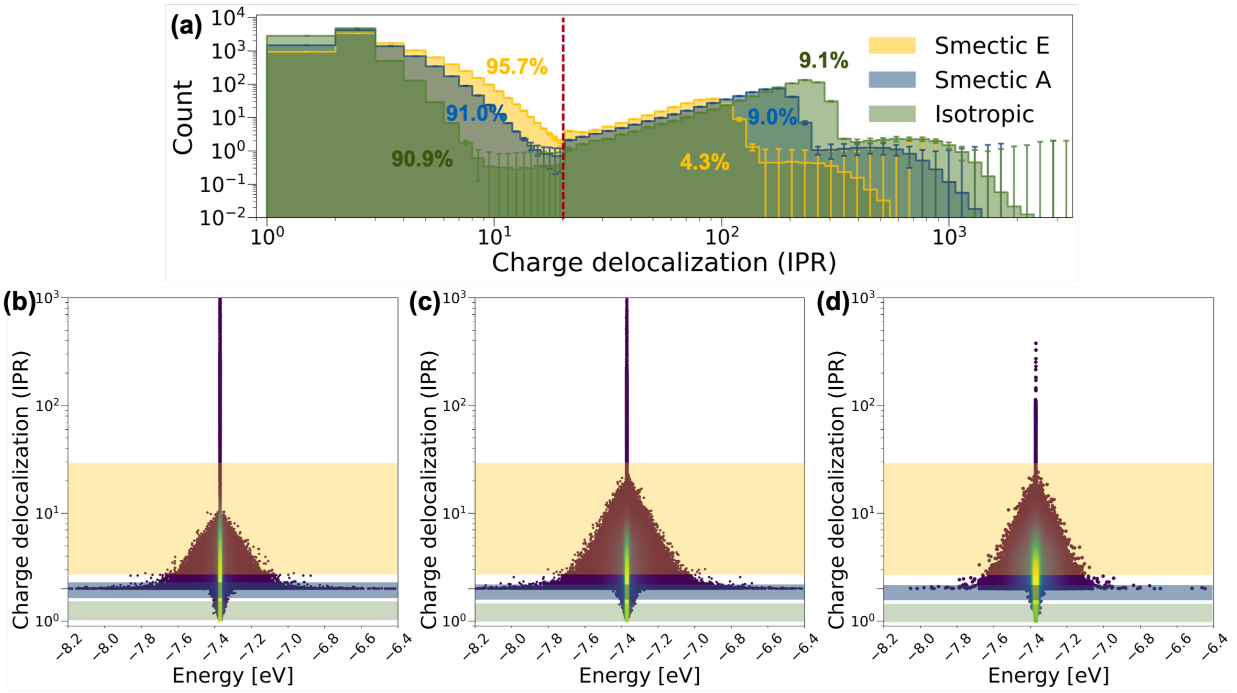

Figure S17: (a) Histograms depicting the number of charge-delocalized molecules (IPR value) for the 9,000 CT states in the cases of constant onsite energy, and the corresponding IPR value distribution (indicating the degree of charge delocalization) plotted against electronic state energy across 9,000 electronic states for (b) the isotropic phase, (c) smectic A phase and smectic E, where the orange, blue, and green masks highlight electronic states with IPR>3, IPR=2, and IPR=1, respectively. The heatmap provides a visual representation of the data point density. The Histograms represent averages over the 2,400 Hamiltonians for each LC phase.

S7 Challenges of Bulk Structural Characterization: Insights from Local Nematic Order and IPR Correlations

In the main text, we emphasize that bulk structural characterizations can overlook the contribution of specific molecular pairs. Here, we provide additional examples by examining the correlation between the Inverse Participation Ratio (IPR), a measure of wavefunction delocalization, and the local nematic order parameter to further illustrate the pitfalls induced by local structural heterogeneity. Figure S18 or Fig. 2c in the main content demonstrates the local nematic order parameter as a function of the characteristic radius in both LC phases. It reveals that the mean value and standard deviation of the order parameter with a characteristic radius smaller than 20 Å (approximately corresponding to three molecular shells) can be much larger than the order parameter with a characteristic radius greater than 50 Å. The substantial standard deviation indicates a significant variation in the local molecular environment, implying the presence of some extremely ordered or disordered local structures. These extreme "outliers" typically play a significant role in charge transport mechanisms but can be easily averaged out by bulk (i.e. field-based) characterizations.

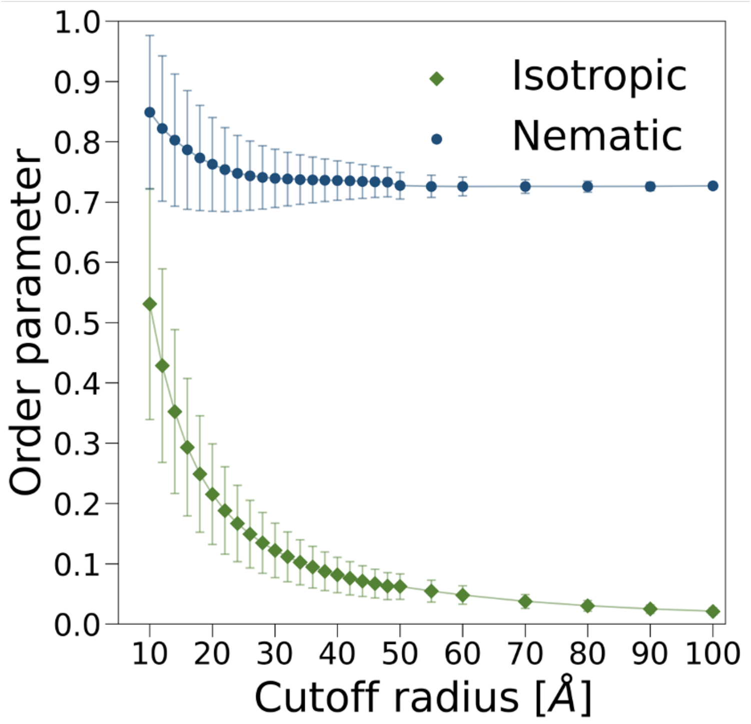

Figure S18: Local nematic order parameter as a function of characteristic radius for isotropic and smectic A phases.

S8 Rejection-Free Kinetic Monte Carlo Algorithm for Charge Transpor Simulation

We employed rejection-free kinetic Monte Carlo (kMC) simulations to model charge diffusion trajectories through BTBT in each LC phase. Generating kMC charge hopping trajectories with 10^5 steps for each LC phase, we utilize a Hamiltonian specific to the corresponding CG configuration. At each KMC step with the carrier localized at site i, the next hop site j is determined from the pool of connected sites with a probability proportional to the rate k_{ij} associated with each connection. This selection process involves picking a random number R between 0 and the sum of the rates $\sum_j k_{ij}$ for all sites connected with nonzero rates to the current site i. Subsequently, the waiting times Δt for the selected process to occur are determined by $(\sum_j k_{ij})^{-1} ln(1/R')$, where R' is another random number between 0 and 1. Once a process and waiting time are selected, a KMC cycle involves moving the carrier to the new site and repeating the process. Further details of the kMC algorithm can be found in Refs. S15–S17.

In this study, the hopping rate from site i to site j (k_{ij}) is determined by

$$k_{ij} = \frac{2\pi}{\hbar} \frac{H_{ij}^2}{\sqrt{4\pi\lambda k_{\mathrm{B}}T}} \exp\left[-\frac{(\Delta E_{ij} - \lambda)^2}{4\pi\lambda k_{\mathrm{B}}T}\right], \tag{S2}$$

where H_{ij} represents the electronic coupling between site i and site j, ΔE_{ij} denotes the HOMO energy difference ($H_{jj} - H_{ii}$), and λ denotes the inner-sphere reorganization energy, with a value of 0.430 eV obtained by the four-point method using the geometry-optimized BTBT.[S15,S18] The outer-sphere reorganization energy is neglected in this work. In Eq. S2, $\hbar$ and k_{B} represent Planck and Boltzmann constants, respectively. For each LC phase, KMC charge hopping trajectories were generated with 10^5 moves based on 100 Hamiltonians derived from CG configurations, utilizing 9,000 molecules as different initial hopping sites, resulting in 900,000 KMC trajectories per LC phase.

With the KMC trajectories, zero-field mobilities were computed from the mean-square displacements (MSD) of the charge carriers along the three Cartesian coordinate directions. Following the Einstein-Smoluchowski relation, the mobility μ along a direction $\mathbf{u}$ is proportional to the charge diffusion coefficient D and is defined as

$$\mu(\mathbf{u}) = \frac{eD}{k_{\mathrm{B}T}} = \frac{e}{k_{\mathrm{B}T}} \lim_{t\to\infty} \frac{1}{2t} \left\langle ([\mathbf{r}(t) - \mathbf{r}(0)] \cdot \mathbf{u})^2 \right\rangle, \tag{S3}$$

where e represents the elementary charge and $\mathbf{r}(t)$ denotes the coordinate of the diffusing charge at time t. Here, $\mathbf{u}$ was defined as (1,0,0), (0,1,0), and (0,0,1) to characterize the mobility along the three Cartesian axes. The angular brackets denote an average over 9,000 independent KMC simulations using 9,000 BTBT molecules within a CG configuration as initial hopping sites. The reported mobilities along the three axes (μ_x, μ_y, and μ_z) are then averaged over 100 CG configurations.

S9 Correlation between IPR and Field-Based Descriptors

To probe the potential feasibility of field-based CG descriptions of electronic structure, we investigated the correlation between IPR and various field-based descriptors. In the smectic A phase (or smectic E phase), as illustrated in Fig.S20 (or Fig.S21), we observed weak correlations, such as a subtle visual link between high IPR values and elevated local nematic order parameters. However, in the isotropic phase (Fig.S19), no linear correlation between IPR and the field-based descriptors was identified. These observation are further supported by quantitative estimates from the Least Absolute Shrinkage and Selection Operator (LASSO) regression, indicating negligible linear correlation (all correlation coefficient elements close to zero) for both LC phases. While a linear relationship between electronic properties and structural descriptors is absent, the possibility of a correlation in nonlinear space remains

an avenue beyond the current scope of our work.

Regarding the Steinhardt order parameter, its general form is defined as Eq. S4 and S5, which is often used to determine crystal structures in molecular simulations.[S19–S21] First, the $q_{lm}(i)$ takes the average of the spherical harmonics Y_{lm} based on the vector $\mathbf{r}_{ij}$ between the reference i_{th} molecule and its the j_{th} molecule among the N_{adj} neighbors of the reference. Then, $q_l(i)$ for a particular atom or point i is calculated as the square root of the sum of the squared absolute values of q_{lm} over all magnetic quantum numbers m from $-l$ to l. The sum is then normalized by a factor involving l in the denominator.

$$q_l(i) = \sqrt{\frac{4\pi}{2l+1} \sum_{m=-l}^{l} |q_{lm}(i)|^2} \tag{S4}$$

where

$$q_{lm}(i) = \frac{1}{N_{\mathrm{adj}}(i)} \sum_{j=1}^{N_{\mathrm{adj}}(i)} Y_{lm}(\mathbf{r}_{ij}) \tag{S5}$$

The averaged 4-fold (6-fold) Steinhardt order parameter in this study is computed with $l = 4$ ($l = 6$), utilizing the center of mass of all BTBT molecules for the calculation, and subsequently obtaining the average values. In crystal units, the 6-fold Steinhardt order parameter typically ranges from 0.7 to 1.0, while the 4-fold Steinhardt order parameter varies from 0 to 1, depending on the crystal type.[S19–S21] For the liquid phase, both the 4-fold and 6-fold Steinhardt order parameters are consistently smaller than 0.4.[S19–S21] The averaged 4-fold and 6-fold Steinhardt order parameters of BTBT in each LC phase fall within this general range. Interestingly, we observe a decreasing trend from the isotropic to the smectic phase, a pattern that aligns with the findings from a recent AA MD simulation of a BTBT derivative.[S22]

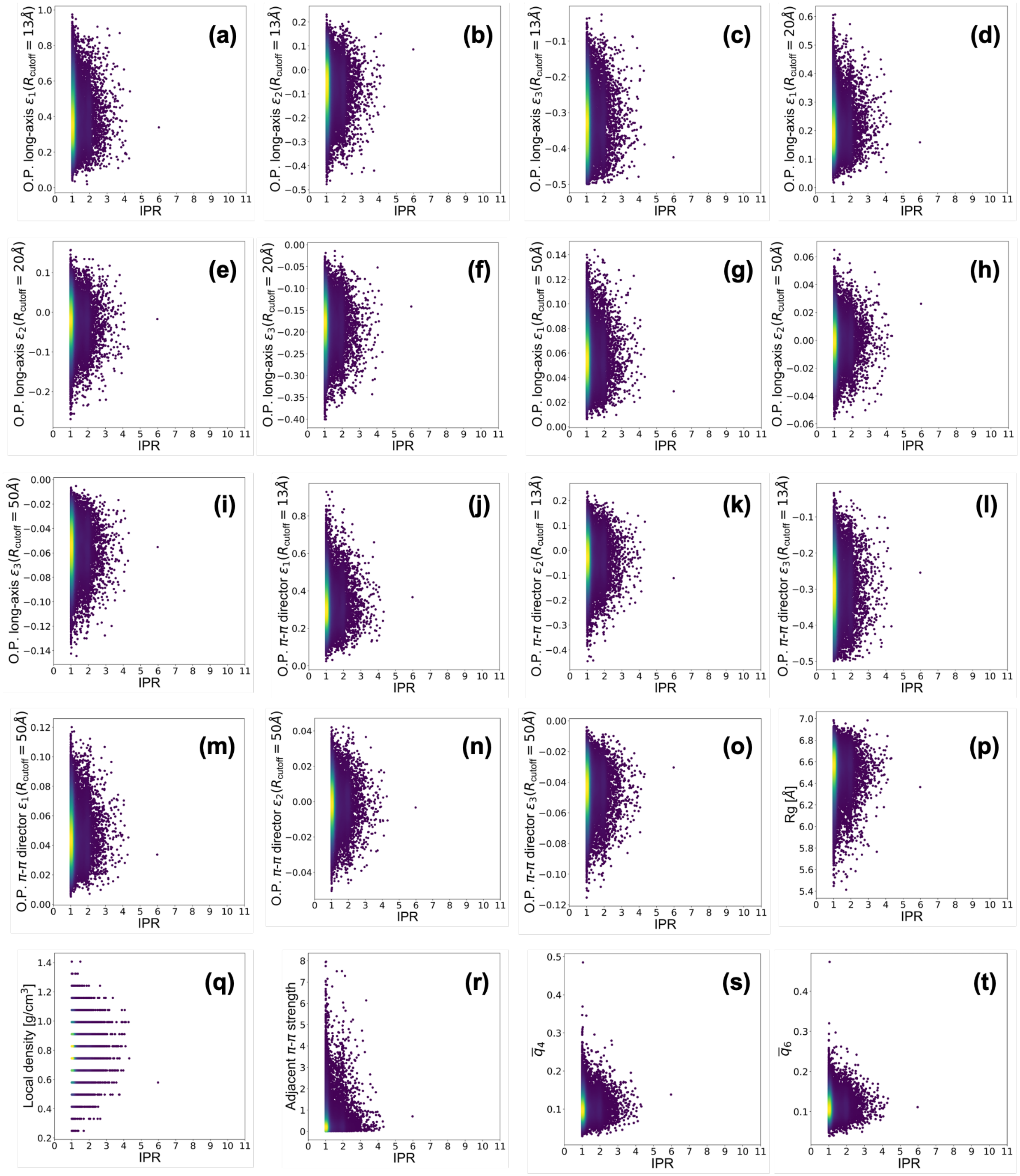

Figure S19: Correlation between IPR and structural descriptors in the isotropic phase: (a-c) eigenvalues of nematic order parameter based on the long-axis of BTBT molecules within a characteristic radius of 13Å, (d-f) eigenvalues of nematic order parameter based on the long-axis of BTBT molecules within a characteristic radius of 20Å, (g-i) eigenvalues of nematic order parameter based on the long-axis of BTBT molecules within a characteristic radius of 50Å, (j-l) eigenvalues of nematic order parameter based on the $\pi - \pi$ director of BTBT molecules within a characteristic radius of 20Å, (m-o) eigenvalues of nematic order parameter based on the $\pi - \pi$ director of BTBT molecules within a characteristic radius of 50Å, (p) radius of gyration, (q) local density, (r) adjacent $\pi - \pi$ stacking strength, and (s) the 4-fold and (t) 6-fold Steinhardt order parameters. The heatmap illustrates the data point density.

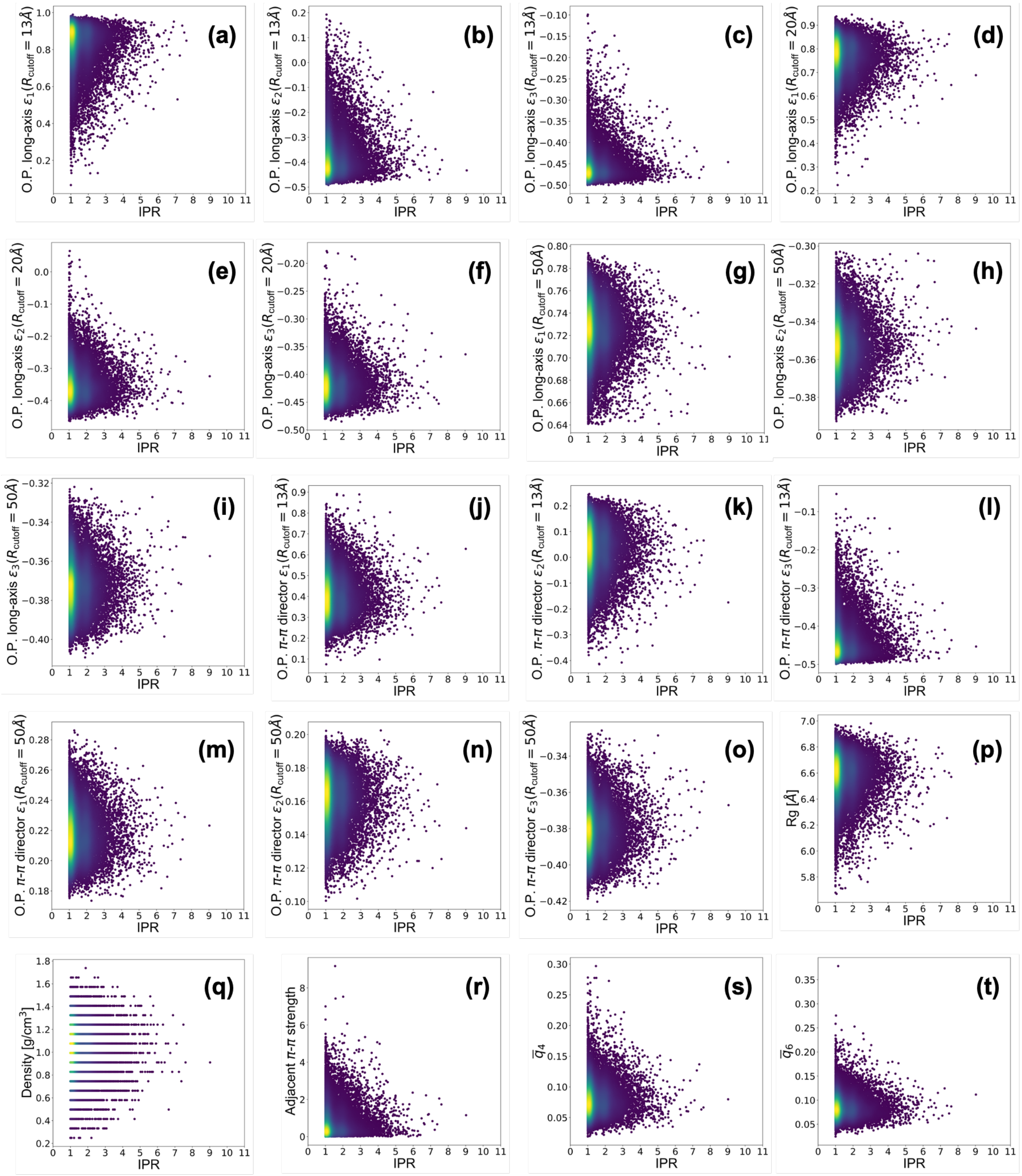

Figure S20: Correlation between IPR and structural descriptors in the smectic A phase: (a-c) eigenvalues of nematic order parameter based on the long-axis of BTBT molecules within a characteristic radius of 13Å, (d-f) eigenvalues of nematic order parameter based on the long-axis of BTBT molecules within a characteristic radius of 20Å, (g-i) eigenvalues of nematic order parameter based on the long-axis of BTBT molecules within a characteristic radius of 50Å, (j-l) eigenvalues of nematic order parameter based on the $\pi - \pi$ director of BTBT molecules within a characteristic radius of 20Å, (m-o) eigenvalues of nematic order parameter based on the $\pi - \pi$ director of BTBT molecules within a characteristic radius of 50Å, (p) radius of gyration, (q) local density, (r) adjacent $\pi - \pi$ stacking strength, and (s) the 4-fold and (t) 6-fold Steinhardt order parameters. The heatmap illustrates the data point density.

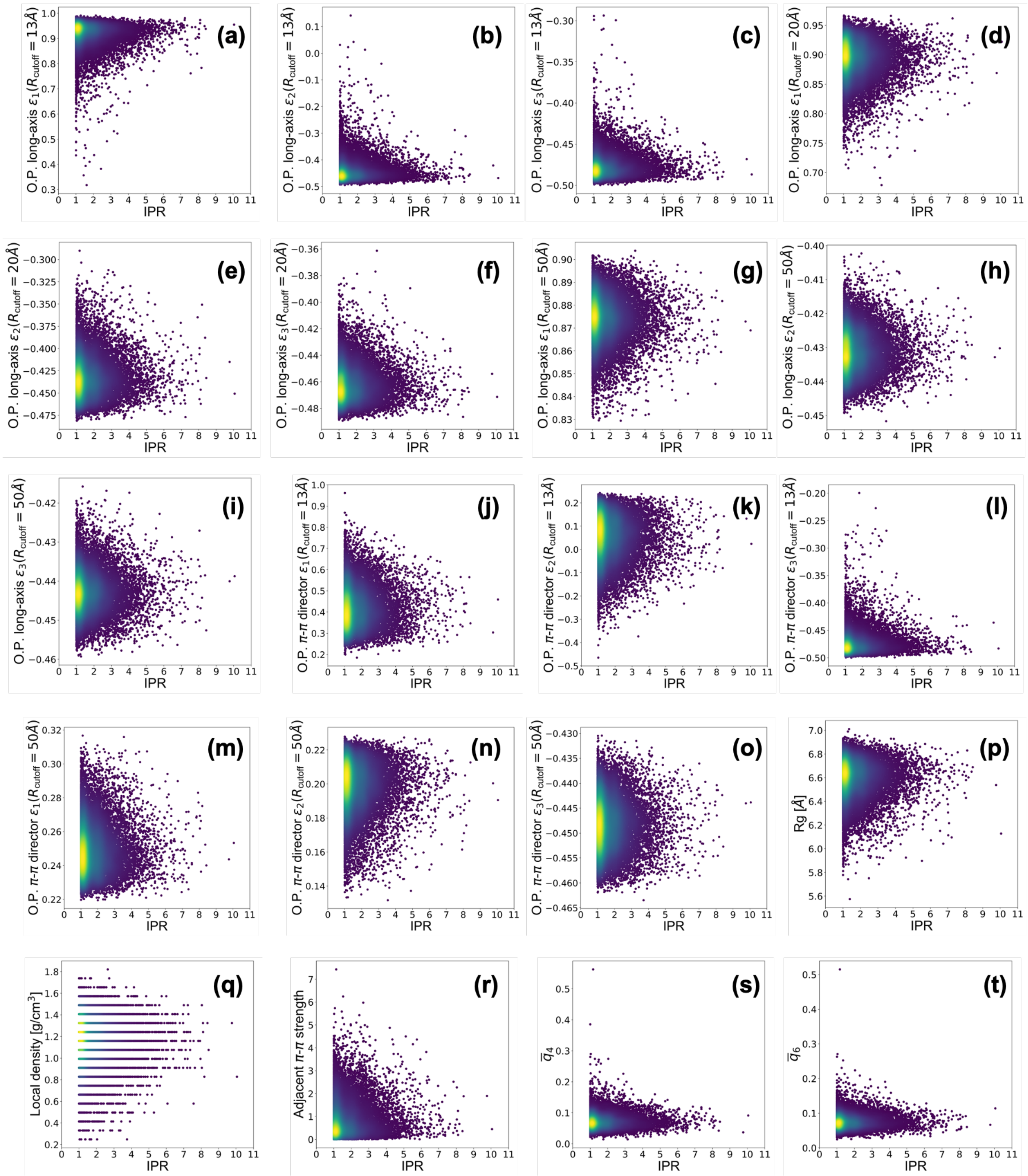

Figure S21: Correlation between IPR and structural descriptors in the smectic E phase: (a-c) eigenvalues of nematic order parameter based on the long-axis of BTBT molecules within a characteristic radius of 13Å, (d-f) eigenvalues of nematic order parameter based on the long-axis of BTBT molecules within a characteristic radius of 20Å, (g-i) eigenvalues of nematic order parameter based on the long-axis of BTBT molecules within a characteristic radius of 50Å, (j-l) eigenvalues of nematic order parameter based on the $\pi - \pi$ director of BTBT molecules within a characteristic radius of 20Å, (m-o) eigenvalues of nematic order parameter based on the $\pi - \pi$ director of BTBT molecules within a characteristic radius of 50Å, (p) radius of gyration, (q) local density, (r) adjacent $\pi - \pi$ stacking strength, and (s) the 4-fold and (t) 6-fold Steinhardt order parameters. The heatmap illustrates the data point density.

References

(S1) William L. Jorgensen, a.; Maxwell, D. S.; Tirado-Rives, J. Development and Testing of the OPLS All-Atom Force Field on Conformational Energetics and Properties of Organic Liquids. *J. Am. Chem. Soc.* **1996**, *1996*, ,45.

(S2) Wang, C.-I.; Maier, J. C.; Jackson, N. E. Identifying Coarse-Grained Representations for Electronic Predictions. *J. Chem. Theory Comput.* **2023**, *19*, 4982–4990.

(S3) Thompson, A. P.; Aktulga, H. M.; Berger, R.; Bolintineanu, D. S.; Brown, W. M.; Crozier, P. S.; in 't Veld, P. J.; Kohlmeyer, A.; Moore, S. G.; Nguyen, T. D.; Shan, R.; Stevens, M. J.; Tranchida, J.; Trott, C.; Plimpton, S. J. LAMMPS - a flexible simulation tool for particle-based materials modeling at the atomic, meso, and continuum scales. *Comp. Phys. Comm.* **2022**, *271*, 108171.

(S4) Soper, A. K. Empirical potential Monte Carlo simulation of fluid structure. *Chem. Phys.* **1996**, *202*, 295–306.

(S5) Reith, D.; Pütz, M.; Müller-Plathe, F. Deriving effective mesoscale potentials from atomistic simulations. *J. Comput. Chem.* **2003**, *24*, 1624–1636.

(S6) Wang, H.; Junghans, C.; Kremer, K. Comparative atomistic and coarse-grained study of water: What do we lose by coarse-graining? *Eur. Phys. J. E* **2009**, *28*, 221–229.

(S7) Dhamankar, S.; Webb, M. A. Chemically specific coarse-graining of polymers: Methods and prospects. *J. Polym. Sci.* **2021**, *59*, 2613–2643.

(S8) Jin, J.; Pak, A. J.; Durumeric, A. E. P.; Loose, T. D.; Voth, G. A. Bottom-up Coarse-Graining: Principles and Perspectives. *J. Chem. Theory Comput.* **2022**, *18*, 5759–5791.

(S9) Noid, W. G. Perspective: Advances, Challenges, and Insight for Predictive Coarse-Grained Models. *J. Phys. Chem. B* **2023**, *127*, 4174–4207.

(S10) Ohta, K.; Closs, G. L.; Morokuma, K.; Green, N. J. Stereoelectronic effects in intramolecular long-distance electron transfer in radical anions as predicted by ab-initio MO calculations. *J. Am. Chem. Soc.* **1986**, *108*, 1319–1320.

(S11) Senthilkumar, K.; Grozema, F. C.; Bickelhaupt, F. M.; Siebbeles, L. D. A. Charge transport in columnar stacked triphenylenes: Effects of conformational fluctuations on charge transfer integrals and site energies. *J. Chem. Phys.* **2003**, *119*, 9809–9817.

(S12) Valeev, E. F.; Coropceanu, V.; da Silva Filho, D. A.; Salman, S.; Brédas, J.-L. Effect of Electronic Polarization on Charge-Transport Parameters in Molecular Organic Semiconductors. *J. Am. Chem. Soc.* **2006**, *128*, 9882–9886.

(S13) Wang, C.-I.; Braza, M. K. E.; Claudio, G. C.; Nellas, R. B.; Hsu, C.-P. Machine Learning for Predicting Electron Transfer Coupling. *J. Phys. Chem. A* **2019**, *123*, 7792–7802.

(S14) Wang, C.-I.; Joanito, I.; Lan, C.-F.; Hsu, C.-P. Artificial neural networks for predicting charge transfer coupling. *J. Chem. Phys.* **2020**, *153*, 214113.

(S15) Brédas, J.-L.; Beljonne, D.; Coropceanu, V.; Cornil, J. Charge-Transfer and Energy-Transfer Processes in π-Conjugated Oligomers and Polymers: A Molecular Picture. *Chem. Rev.* **2004**, *104*, 4971–5004.

(S16) Rühle, V.; Lukyanov, A.; May, F.; Schrader, M.; Vehoff, T.; Kirkpatrick, J.; Baumeier, B.; Andrienko, D. Microscopic Simulations of Charge Transport in Disordered Organic Semiconductors. *J. Chem. Theory Comput.* **2011**, *7*, 3335–3345.

(S17) Oberhofer, H.; Reuter, K.; Blumberger, J. Charge Transport in Molecular Materials: An Assessment of Computational Methods. *Chem. Rev.* **2017**, *117*, 10319–10357.

(S18) Nelsen, S. F.; Blackstock, S. C.; Kim, Y. Estimation of inner shell Marcus terms

for amino nitrogen compounds by molecular orbital calculations. *J. Am. Chem. Soc.* **1987**, *109*, 677–682.

(S19) Steinhardt, P. J.; Nelson, D. R.; Ronchetti, M. Bond-orientational order in liquids and glasses. *Phys. Rev. B* **1983**, *28*, 784–805.

(S20) Lechner, W.; Dellago, C. Accurate determination of crystal structures based on averaged local bond order parameters. *J. Chem. Phys.* **2008**, *129*, 114707.

(S21) Ramasubramani, V.; Dice, B. D.; Harper, E. S.; Spellings, M. P.; Anderson, J. A.; Glotzer, S. C. freud: A Software Suite for High Throughput Analysis of Particle Simulation Data. *Computer Physics Communications* **2020**, *254*, 107275.

(S22) Baggioli, A.; Casalegno, M.; Raos, G.; Muccioli, L.; Orlandi, S.; Zannoni, C. Atomistic Simulation of Phase Transitions and Charge Mobility for the Organic Semiconductor Ph-BTBT-C10. *Chem. Mater.* **2019**, *31*, 7092–7103.
